# Epigenetic regulation in major depression and other stress-related disorders: molecular mechanisms, clinical relevance and therapeutic potential

**DOI:** 10.1038/s41392-023-01519-z

**Published:** 2023-08-30

**Authors:** Minlan Yuan, Biao Yang, Gerson Rothschild, J. John Mann, Larry D. Sanford, Xiangdong Tang, Canhua Huang, Chuang Wang, Wei Zhang

**Affiliations:** 1 Mental Health Center and Psychiatric Laboratory, the State Key Laboratory of Biotherapy, West China Hospital, Sichuan University, Chengdu, 610041 China; 2 Department of Abdominal Oncology, West China Hospital, Sichuan University, Chengdu, 610041 China; 3 Department of Microbiology and Immunology, Vagelos College of Physicians and Surgeons, Columbia University, New York, NY 10032 USA; 4 Department of Psychiatry, Columbia University, New York, NY 10032 USA; 5 Molecular Imaging and Neuropathology Division, New York State Psychiatric Institute, New York, NY 10032 USA; 6 Department of Radiology, Columbia University, New York, NY 10032 USA; 7 Sleep Research Laboratory, Center for Integrative Neuroscience and Inflammatory Diseases, Pathology and Anatomy, Eastern Virginia Medical School, Norfolk, VA USA; 8 Sleep Medicine Center, Department of Respiratory and Critical Care Medicine, Mental Health Center, Translational Neuroscience Center, and State Key Laboratory of Biotherapy, West China Hospital, Sichuan University, Chengdu, 610041 China; 9 Department of Biotherapy, Cancer Center and State Key Laboratory of Biotherapy, West China Hospital, Sichuan University, Chengdu, 610041 China; 10 Department of Pharmacology, and Provincial Key Laboratory of Pathophysiology in School of Medicine, Ningbo University, Ningbo, Zhejiang 315211 China; 11 West China Biomedical Big Data Center, West China Hospital, Sichuan University, Chengdu, 610041 China; 12 Medical Big Data Center, Sichuan University, Chengdu, 610041 China

**Keywords:** Epigenetics in the nervous system, Epigenetics

## Abstract

Major depressive disorder (MDD) is a chronic, generally episodic and debilitating disease that affects an estimated 300 million people worldwide, but its pathogenesis is poorly understood. The heritability estimate of MDD is 30–40%, suggesting that genetics alone do not account for most of the risk of major depression. Another factor known to associate with MDD involves environmental stressors such as childhood adversity and recent life stress. Recent studies have emerged to show that the biological impact of environmental factors in MDD and other stress-related disorders is mediated by a variety of epigenetic modifications. These epigenetic modification alterations contribute to abnormal neuroendocrine responses, neuroplasticity impairment, neurotransmission and neuroglia dysfunction, which are involved in the pathophysiology of MDD. Furthermore, epigenetic marks have been associated with the diagnosis and treatment of MDD. The evaluation of epigenetic modifications holds promise for further understanding of the heterogeneous etiology and complex phenotypes of MDD, and may identify new therapeutic targets. Here, we review preclinical and clinical epigenetic findings, including DNA methylation, histone modification, noncoding RNA, RNA modification, and chromatin remodeling factor in MDD. In addition, we elaborate on the contribution of these epigenetic mechanisms to the pathological trait variability in depression and discuss how such mechanisms can be exploited for therapeutic purposes.

## Introduction

Major depressive disorder (MDD) affects an estimated 300 million people worldwide.^1^ The condition is characterized by episodes of low mood, anhedonia or loss of interest, feelings of guilt or worthlessness, suicidal thoughts, psychomotor retardation or agitation, impaired cognitive function, and physical symptoms such as changes in appetite and disrupted sleep patterns.^2,3^ It has become one of the leading cause of disease burden worldwide,^4–6^ impairs human capability in terms of education, employment and relationships, and is also linked to premature mortality from suicide and other diseases.^7–10^ It has been reported that the prevalence and burden of depressive and anxiety disorders have increased dramatically worldwide (more than 25% during the first year of the pandemic) during the COVID-19 pandemic,^11^ thus posing grave challenges for mental health services during and after the epidemic.^12^ To date, causal mechanisms and pathogenesis of MDD are only partly understood. The heritability of MDD is estimated to be about 35%,^13^ which is lower than estimates of genetic contributions to other psychiatric disorders like schizophrenia and bipolar disorder (with heritability rates thought to be 65–70%).^14^ Genome-wide association studies (GWAS) have recently identified over 80 reproducible loci contributing to MDD, each with only a small effect.^15,16^ Moreover, the variance explained by major depression polygenic risk scores based on these genomic loci is still a very low fraction of the total heritable risk.^15^ These findings suggested that we are yet to discover most gene variants contributing to the genetic risk and that genetics alone do not account for most of the risk of major depression.^17^

Epidemiological studies indicate that environmental factors are strongly associated with the risk of developing MDD and other stress-related disorders.^18–22^ Early studies examined how stressful life experiences affected MDD, usually in the year preceding its onset.^23,24^ These documented stressful events occur mainly in adulthood. They include bereavement, financial crisis, loss of employment, separation, academic setbacks, life-threatening or chronic health problems, persistent physical pain, and exposure to violence.^25^ Adverse experiences early in life, such as maternal stress during pregnancy and poor maternal care after childbirth, childhood physical and sexual abuse, emotional neglect, bullying, or early separation from parents, are associated with subsequent onset, severity and chronicity of MDD.^26,27^ Epigenetics is a molecular mechanism that has attracted attention as it helps explain the biological impact of environmental factors.^28,29^

Epigenetics refers to short- and long-term gene expression variations that are caused by non-DNA-encoded mechanisms.^30,31^ These mechanisms include DNA methylation or hydroxymethylation, chemical changes occurring on histone proteins (histone modification), expression of noncoding RNAs, chromatin remodeling, and RNA modification. These interconnected mechanisms can mold how a cell responds at a molecular level.^31,32^ Epigenetic regulation mediates direct epigenetic effects or gene-by-environment interactions and can lead to complex diseases.^33,34^ The importance of epigenetic alterations and their effects on almost every biological pathway involved in the pathophysiology of MDD and other stress-related disorders, such as anxiety disorders and post-traumatic stress disorder (PTSD) is increasingly appreciated.^35–37^ Epigenetics can regulate neuronal plasticity and memory consolidation.^38–41^ Epigenetic regulation plays a mediating role for abnormal stress response systems, monoamine neurotransmitter dysfunction and neuroinflammation in MDD, and other stress-related disorders in animal models.^42,43^

**Fig. 1:**
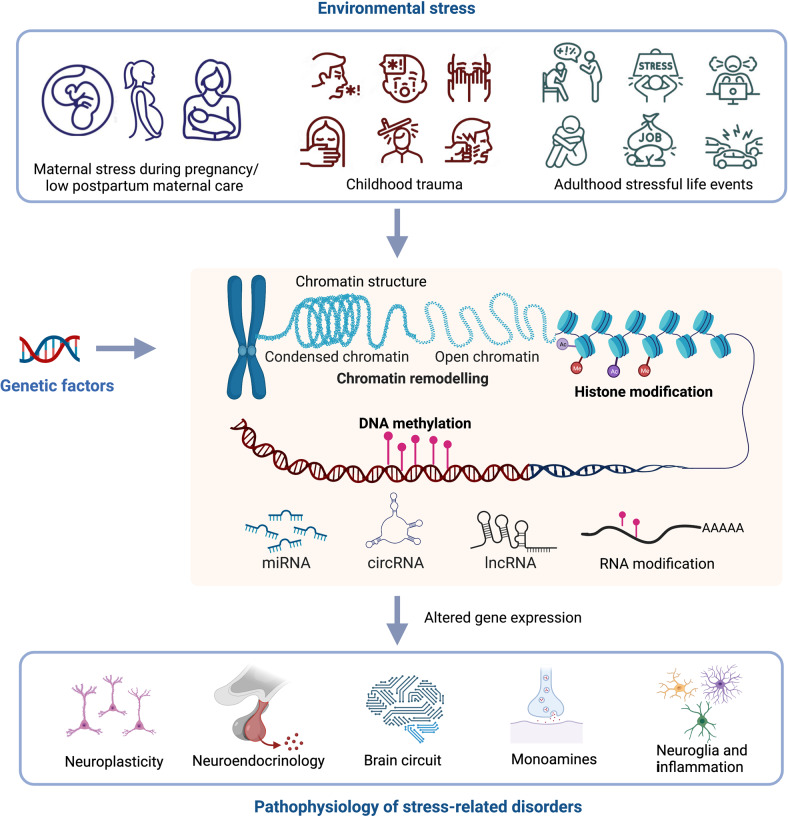
Overview of the role of epigenetic processes on the pathophysiology of stress-related disorders. Environmental factors, including early life stress, childhood trauma, and stressful life events in adulthood contribute to the development of stress-related disorders through direct epigenetic regulation or gene-by-environment interactions. Epigenetic mechanisms include chromatin structure changes, histone modifications, DNA modification, noncoding RNA changes, and RNA modifications. These epigenetic processes play crucial roles in different aspects of the pathophysiology of stress-related disorders

In this review, we first provide an overview of our current understanding of the functional role of different types of epigenetic regulation, including DNA methylation, histone modification, noncoding RNAs and some newly studied modifications such as RNA modification and chromatin structure remodeling factor in stress-related disorders (Fig. 1). Specifically, we discuss the roles of these epigenetic alterations in MDD pathophysiology, including neuroplasticity, neuroendocrinology, neurotransmission, and neuroinflammation. We explain how these epigenetic mechanisms might facilitate diagnosis and treatment of MDD.

## DNA methylation regulates MDD progression

**Fig. 2:**
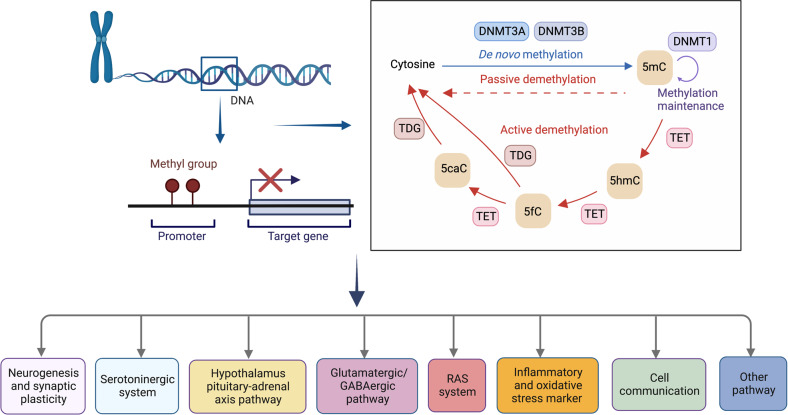
DNA methylation is involved in the progression of MDD and stress-related disorders. DNA methylation is a biological process by which methyl groups are added to the DNA at position 5′ in cytosine (5mC), which is mainly found at CpG dinucleotides. In contrast to DNA methylation, which is set up by methyltransferases (DNMT3A and DNMT3B) and maintained by DNMT1, 5mC is oxidized to 5hmC by the ten-eleven translocation (TET) family of dioxygenase proteins. In successive steps, TET enzymes further hydroxylate 5hmC to 5fC and then 5caC, which are recognized and removed by TDG, generating an unmodified cytosine. Many clinical and animal studies have examined DNA methylation of genes involved in multiple biological pathways. 5mC methylation at position 5′ in the cytosine, DNMT DNA methyltransferase, TET ten-eleven translocation, 5hmC 5-hydroxymethylcytosine, 5fC 5-formylcytosine, 5caC 5-carboxylcytosine, TDG thymine-DNA glycosylase

DNA methylation is the covalent addition of a methyl group to DNA’s cytosine residues, resulting in a methylcytosine (mC) base. In the human genome, mC most frequently occurs at CpG sites (cytosine followed by a guanine base in the DNA sequence).^44,45^ In addition, cytosines followed by a non-guanine base, such as cytosine, adenine, or thymine, might also experience DNA methylation. In brain tissues, such non-CpG methylation is a common alteration that increases in frequency during development.^46^ DNA methyltransferases (DNMTs) include DNMT1, DNMT2, DNMT3A, DNMT3B, and DNMTL (Fig. 2).^47,48^ Methylated DNA triggers a recognition response by proteins known as methyl-CpG-binding domain (MBD) proteins, which lead to the recruitment of other proteins that either activate or repress gene expression. Methyl-CpG binding protein 2 (MeCP2) and methyl-CpG-binding domain 1 (MBD1) are two examples of MBD proteins that detect methylated DNA and are known for their association with neurodevelopment.^49–51^ DNA methylation is widely recognized as the most extensively researched epigenetic mechanism and is generally believed to be stable over the course of an organism’s lifetime.^52^

### The location and functional effects of DNA methylation

It is often postulated that increased methylation of CpG islands in promoter regions results in the suppression of gene expression, and decreased methylation leads to increased gene expression. For example, increased CpG methylation in the promoter region of the gene encoding brain-derived neurotrophic factor (BDNF) has been found to correlate with a decreased synthesis of BDNF in neurons.^53^ However, this only reliably occurs in the promoter region surrounding the first exon. For other genomic locations, this is different. One study showed that the methylation of non-proximal promoters, which is dependent on DNMT3a, enhances the expression of a large cohort of neurogenic genes.^54^ Another study demonstrated a positive correlation between gene expression and gene body methylation.^55^ In clinical studies, variable associations of DNA methylation and MDD have been shown to occur in regions outside the promoter. One example showed hypomethylation of synapsins (SYN2) linked to depression.^56^ In contrast, a study found that patients with MDD had increased methylation in the *TESC* gene, negatively correlated with the right parahippocampal cingulum integrity.^57^ These studies suggest that the association between methylation changes and MDD is diverse, with no common effects throughout the genome or specific genomic locations.

Methylation adds to the diversity of genomic responses because DNA methylation impairs access of transcription factors to gene regulatory regions. When a recruited transcription factor is inhibitory, DNA methylation results in enhanced gene expression.^58^ Another possible reason for different transcriptional effects of DNA modifications may be the presence of variant forms of 5-methylcytosine, such as 5-hydroxymethylcytosine, which is produced by the addition of hydroxyl groups to 5-methylcytosine under the action of the ten-eleven translocation (TET) enzymes (Fig. 2).^59,60^ Currently, the majority of reports in the literature do not differentiate between DNA methylation and hydroxymethylation, and assays that rely on bisulfite conversion measure both modifications indiscriminately. Future studies must investigate these two changes in parallel to provide more information.

Non-CpG methylation is highly concentrated in neurons and glial cells^61^ and accumulates in neurons as they mature.^62^ It is a rare occurrence in the frontal cortex of human fetuses; however, it increases significantly during later stages of life. This increase in non-CpG methylation is accompanied by the development of synapses and increased synaptic density.^61^ As more research is conducted, it becomes increasingly evident that non-CpG methylation plays a significant role in regulating gene activity and can continue to function in the adult brain, where it may act similarly to CpG methylation in repressing transcription.^63^ A previous study showed that the binding of MeCP2 to non-CpG methylates DNA sequences is critical for BDNF expression. This process influenced the onset timing for Rett syndrome.^64^ Although the importance of non-CpG methylation in the nervous system has been demonstrated, whether it contributes to the pathophysiology of MDD and other stress-related disorders remains poorly investigated and requires further study.

### Sensitive periods of DNA methylation in stress vulnerability

Exposure to environmental factors such as stress, toxins, or viruses at particularly vulnerable times of fetal development or early infancy may predispose the body to diseases in adulthood.^65^ Some of these effects may be mediated by epigenetic mechanisms.^66,67^ Early stages of life, from embryonic development through adolescence, include the ages during which the development and the later plasticity capacity of neuronal circuits are formed, along with immune, stress response, and hormone regulation pathways. These early stages are a time window when there is greater susceptibility to environmental toxins than at later periods in life.^68–70^

Previous studies undertaken in the rat explored potential mechanisms to explain how maternal care practice variations impact the development of individual variances in the stress response.^71–74^ Female Long-Evans rats differ significantly in how frequently they lick/groom (LG) their pups, which is a stable feature of the maternal phenotype. When compared to animals raised by high LG mothers, the adult offspring from low LG mothers had less hippocampal glucocorticoid receptor (GR) and protein expression, lower plasma pituitary adrenocorticotropin (ACTH) and impaired corticosterone responses to acute stress,^71,74,75^ and was more vulnerable to show learned helplessness to environmental stress.^76^ In the hippocampus of adult rats with low maternal care, the transcription factor nerve growth factor-inducible factor A (NGFI-A) binding region of the GR promoter 1_7_ gene is hypermethylated, but in those with high maternal care, it is hypomethylated. Cross-fostering reverses these methylation discrepancies.^74^ However, when the timing of the stressor was shifted to adulthood, there was little effect on the GR promoter methylation levels in the brain (neither in the hippocampus or hypothalamic paraventricular nucleus).^77^ However, an increase in methylation levels in the peripheral hypothalamic-pituitary-adrenal (HPA) axis tissues was found to be accompanied by chronic stress.^77^ The findings of these animal studies illustrate how DNA methylation is affected by the timing of stress relative to sensitive periods.

Studies using human postmortem tissues also showed a link between early life adversity and epigenetic regulation of GR expression in the hippocampus. Lower GR expression along with higher levels of cytosine methylation of the GR promoter exon 1 F have been reported in suicide decedents with a history of childhood abuse than in suicide decedents without a history of abuse as well as in non-suicide controls.^78^ Cell type-specific alterations in the methylation of DNA in oligodendrocyte genes along with a general disruption of the transcriptional program related to myelin, were reported in depressed suicide decedents with childhood maltreatment.^79^ Impaired myelination in the anterior cingulate cortex in those with childhood abuse was also observed. Furthermore, recent clinical studies demonstrate that the developmental timing of childhood adversity, with sensitive periods before three years of age, explains more variability in DNA methylation than the accumulation or recency of exposure.^39^ This suggests that early childhood is a crucial time when exposure to life stress predicts altered DNA methylation patterns.

### Tissue- and cell-type-specific changes in MDD-associated DNA methylation

#### Tissue specificity

DNA methylation changes in major depression and other stress-related disorders are observed in the brain and other tissues. While brain tissue is usually not available in living human studies, DNA derived from peripheral blood cells, saliva, and cheek swabs is accessible from live subjects. It may enable extensive epigenetic research using samples from various age groups, as well as repeated sampling over time.^80,81^ Whether findings from peripheral tissues are meaningful indicators of the pathogenesis of MDD remains to be delineated. Previous work indicates some overlap between MDD-associated differentially methylated regions (DMRs) in blood and the MDD-associated DMRs in the prefrontal cortex and other brain regions.^82–85^ One study, despite a small sample size, reported that three loci located in GABBR2, RUFY3, and in an intergenic region on chromosome 2, were replicated in blood and some cortical regions (Brodmann area (BA) 10 and 25).^82^ These genes are involved in normal brain development and function.^86^

However, several studies have reported that most DNA methylation markers detected in the peripheral tissues cannot accurately predict the DNA methylation status of the brain.^87^ The latest advancements in array techniques enabled the use of hypothesis-free paradigms to examine the association of DNA methylation changes across the entire genome to study different phenotypes.^88^ Unbiased, genome-wide studies using peripheral blood have reported epigenetic alterations in genes predominantly unassociated with established candidate genes selected based on known pathogenic findings, such as the serotonin transporter gene (SLC6A4) and brain-derived neurotrophic factor (BDNF),^89–93^ indicating alternative or additional pathogenic mechanisms.^94–97^ Another study comparing DNA methylation across the whole genome in live human brain tissue with that in peripheral (blood, saliva, and buccal) tissues concluded that the patterns unique to the target genomic region must be considered when selecting the best surrogate tissue to mimic brain DNA methylation.^98^ Nevertheless, DNA methylation detected in the brain or peripheral tissue could offer instructive insights into different biological pathways implicated in the etiopathogenesis of MDD and other stress-related psychiatric disorders (Fig. 2, Table 1).

**Table 1:** Summary of DNA methylation alterations in depression and related clinical or biological outcomes

Targeted gene (and location)	Candidate or epigenome-wide approach	Sample characteristic (human/animal)	Tissue	Clinical/biological outcome	Study
*Neurogenesis and synaptic plasticity*					
BDNF					
Promoter	Candidate	Human (community residents with and without late-life depression)	Whole blood	↑BDNF DNA methylation (DNAm) → higher depression prevalence and increased depressive severity	^92^
Promoter	Methylome-wide association studies	Human (Monozygotic twins)	Blood (leukocytes)	BDNF, NR3C1, and SLC6A4 DNAm were positively associated with depressive symptoms; BDNF and NR3C1 DNAm mediate the association between childhood trauma and depression.	^93^
Promoter	Candidate	Human (MDD patients and healthy volunteers)	Whole blood	DNAm inversely correlated with the integrity (fractional anisotropy) of the anterior corona radiata in MDD patients.	^431^
Promoter	Candidate	Human (MDD patients and healthy volunteers)	Whole blood	BDNF DNAm mediates the association between neurocognitive performance and two BDNF single nucleotide polymorphisms (SNPs; rs908867 and rs925946).	^432^
Promoter	Candidate	Human (mothers with interpersonal violence-related PTSD)	Saliva-derived DNA	↑BDNF DNAm → maternal anxiety	^433^
PRIMA1					
–	Genome-wide	Human (patients with MDD and matched controls)	Postmortem frontal cortex brain tissue/ lymphoblastoid cell lines	↑PRIMA1 DNAm in MDD → decreased PRIMA1 immunoreactivity for acetylcholinesterase and mRNA levels.	^434^
POU3F1					
–	Genome-wide	Human (suicidal subjects with/without child abuse experience, sudden death controls)	Brian tissue (Anterior cingulate cortex)	Changes in DNAm of oligodendrocyte genes is associated with previous childhood abuse.	^79^
ID3					
Gene body	Genome-wide	Human (children with and without maltreatment)	Saliva-derived DNA	ID3 methylation is correlated with morning cortisol levels in depressive children.	^435^
TPPP					
Gene body	Genome-wide	Human (children with and without maltreatment)	saliva-derived DNA	One CpG site in the gene body of TPPP predicts children’s depression.	^435^
PSD-95 and GJA-1					
–	Candidate	Human (MDD patients and healthy controls)	Brain tissue (prefrontal cortex and hippocampus)	MDD patients did not show differences in PSD-95 and GJA-1 DNA methylation compared with healthy controls.	^97^
TESC					
–	Candidate	Human (MDD patients and healthy controls)	Whole blood	↑TESC gene DNAm → significantly correlated with right PHC integrity in the MDD group.	^57^
SYN2					
Promoter	Candidate	Human (MDD, BD patients and healthy controls)	Brain tissue (BA10)	↓SYN2 DNAm → inversely correlated with SYN2a mRNA expression.	^56^
*Serotoninergic system*					
SLC6A4					
Promoter	Candidate	Human (patients with MDD)	Whole blood	↑SLC6A4 promoter methylation → higher childhood adversities, family history of depression, perceived stress, and the manifestation of more serious psychopathology.	^89^
Promoter, exon1, intron1	Candidate	Human (Adolescent participants from a cohort)	Buccal cell	↑ 5HTT DNAm who carried 5HTTLPR short-allele → more common depressive symptoms.	^90^
Promoter	Candidate	Human (Iowa Adoption Study)	Blood (lymphoblastoid cell lines)	↑DNAm → lifetime history of major depression	^436^
Promoter	Candidate	Human (Monozygotic twins with MDD)	Blood (leukocytes)	↑Mean DNAm → increase in the difference in depressive symptom scores. The 5-HTTLPR genotype does not modulate this association.	^437^
Promoter	Candidate	Human (MDD patients and healthy controls)	Whole blood	Negative emotional content significantly correlated positively with anterior insula activation and SLC6A4 methylation levels.	^438^
Up upstream to the transcription start site	Candidate	Human (MDD patients and healthy controls)	Whole blood	↑SLC6A4 DNAm at CpG2 in MDD → ↓white matter integrity in the corpus callosum.	^439^
First extron/intron	Candidate	Human (PTSD patients and healthy controls)	Whole blood	↓SLC6A4 DNAm and traumatic events → PTSD	^440^
MAO-A					
Promoter and exon1/intron1 region	Candidate	Human (MDD patients)	Whole blood	↓MAO-A promoter DNAm hypomethylation →impaired treatment response in female patients with MDD.	^441^
Promoter	Candidate	Human (MDD patients)	Saliva samples	↓MAO-A DNAm hypomethylation → higher MAO-A expression and depression in female patients.	^442^
First exon region	Candidate	Human (MDD patients)	Saliva samples	↓DNAm → a history of depression; ↑DNAm in female individuals compared to males.	^443^
*Hypothalamus pituitary-adrenal axis pathway*					
NR3C1					
Promoter	Candidate	Human (adult residents)	Whole blood	↓DNAm associated with MDD, ↑DNAm associated with childhood maltreatment.	^444^
Exon 1F	Candidate	Human (MDD patients, healthy controls)	Whole blood	↑NR3C1 exon 1F DNAm → ↑morning cortisol concentrations.	^445^
1F promoter	Candidate	Human (MDD patients, healthy controls)	Whole blood	↓DNAm at two CpG sites in MDD associated with hippocampal subfields.	^446^
Promoter	Candidate	Human (MDD patients, healthy controls) brain	Post-mortem tissues	MDD patients did not show differences in NR3C1 DNAm compared with healthy controls.	^447^
Promoter	Candidate	Animal model (adult rat)	Brain tissue	Females showed ↑NR3C1 DNAm in hippocampus	^448^
Promoter	Candidate	Animal model	Brain tissue	↑Hippocampal NR3C1 DNAm in maternally separated males; ↑hippocampal BDNF IX DNAm in male and female maternally separated mice.	^449^
1F promoter	Candidate	Human (combat veterans/PTSD)	Peripheral blood mononuclear cells (PBMCs)	↓NR3C1 DNAm → glucocorticoid activity, PTSD symptoms↑	^450^
1F promoter	Candidate	Human (generalized anxiety disorder patients, healthy controls)	PBMCs	↑ NR3C1 DNAm negatively correlated with serum basal cortisol levels and GR sensitivity in the PBMCs	^451^
FKBP5					
Intron 7 GR response element region	Candidate	Human (MDD patients, healthy controls)	Whole blood	↓FKBP5 introns DNAm → childhood adversity in MDD patients carrying the high-risk T allele rs1360780; bilaterally higher activation during valence recognition in MDD.	^452^
Intron 7	Candidate	Human (MDD patients, healthy controls)	Whole blood	MDD patients did not show differences in DNAm at FKBP5 intron 7 compared with healthy controls.	^445^
Intron 7	Candidate	Human (general population sample)	Whole blood	FKBP5 methylation levels were not related to FKBP5 transcription levels.	^453^
Intron 7	Candidate	Human (childhood abuse/PTSD)	Whole blood	↓FKBP5 introns DNAm → altered glucocorticoid responsiveness of FKBP5, altered hippocampal volume	^454^
GLU1					
Promoter	Candidate	Human (MDD patients and healthy controls)	Whole blood	↑GLUT1 DNAm → acute phase of MDD and mild insulin resistance	^455^
OXTR					
4 bp proximal to an estrogen receptor binding region	Candidate	Human (women cohort)	Whole blood	OXTR DNAm negatively correlated with postpartum depression (PPD); a PPD specific negative correlation of DNAm with serum estradiol levels.	^456^
Promoter	Candidate	Human (depressed women, healthy controls)	Leukocyte cell	↓OXTR exon 1 DNAm in depressed female patients compared to nondepressed women. Exon 1 DNAm was associated with depressed traits, whereas rs53576 genotype affected exon 2 methylation.	^457^
CpG site -934	Candidate	Human (depressed women, healthy controls)	Whole blood	rs53576 interacts with DNAm in the OXTR gene among women who developed PPD.	^458^
*Glutamatergic/GABAergic pathway*					
GRIN1					
Gene body	Genome-wide methylation	Human (maltreated and healthy children)	Saliva-derived DNA	DNAm changes in GRIN1 predicted depression independently, beyond the maltreatment history effects.	^435^
*RAS systems*					
ACE					
Promoter and exon 1	Candidate	Human (MDD patients and healthy controls)	Blood (leukocyte)/human post mortem brain tissue	DNAm frequency at the ACE promoter inversely correlated with the serum concentrations of cardiovascular disorders risk markers (ICAM-1, E-selectin, and P-selectin) in depressed patients.	^459^
*Inflammatory and oxidative stress marker*					
YOD1					
—	Epigenome-wide association study	Human (community-based elderly participants)	Brain tissue (dorsal lateral prefrontal cortex)	A significant relationship between DNAm at four CpG sites in YOD1 and late-life MDD, and the effects were more strongly related to late-life MDD in men than in women.	^460^
IL-6					
Promoter	Candidate	Human (community-based elderly participants)	Buccal cells	↓IL6 DNAm → current MDD or high depressive symptoms; ↑IL-6 DNAm at the same site → antidepressant use.	^461^
CRP					
–	Candidate	Human (a large community-based sample)	Whole blood	DNAm of CRP associated with global gray matter/cortical volume reduction and widespread white matter tract integrity impairment.	^462^
*Cell communication*					
DEPDC7					
–	Candidate	Human (general monozygotic twins)	Whole blood	↓ cg09090376DNAm in a cotwin → ↑ depressive symptom score.	^463^
*Other pathway*					
–	Methylome-wide association study	Human (Monozygotic twins with a lifetime history of MDD)	Blood (monocytes)	A significant relationship between 39 DMRs, 30 differentially expressed genes and lifetime history of MDD. These DMRs are involved in various processes including synaptic activity, neuropsychiatric disorders, neuronal plasticity and social behavior.	^464^

#### Cell-type specificity

In addition to tissue specificity considerations, cell-type specificity in epigenetic research could provide more precise insights into the molecular pathology of MDD. In mice that were subjected to chronic stress and exhibiting severe depressive behavior, DNMT3A levels were found to be higher in the nucleus accumbens (NAc) than controls.^99^ Studies examining postmortem brain tissue reported lower DNMT1 levels and higher DNMT3B levels in the frontopolar cortex. The study also reported reduced expression of DNMT1 and DNMT3B in the amygdala, and increased expression of DNMT3B in the paraventricular nucleus of depressed suicide decedents.^100^ Therefore, changes in DNMT mRNA expression occurred in specific cells of autopsied brain tissue in depressed suicide decedents. Fluorescence-activated cell sorting detected oligodendrocyte-specific DNA methylation changes in MDD *postmortem*.^79^ Consistent with the consideration of cell-type specificity in preclinical studies, some clinical studies using peripheral blood to examine DNA methylation changes in MDD corrected for cell composition,^101,102^ with the authors focusing mainly on peripheral immune cells implicated in the pathophysiology of psychiatric disorders.^103^

Changes in DNA methylation of particular genes within specific brain cells that are linked to depression are also evident. DNA methylation at the glial cell-derived neurotrophic factor (Gdnf) promoter with distinct epigenetic modulator complexes between depression-susceptible and depression-resistant animals was increased by chronic stress.^104^ A methylation map specific to astrocytes throughout the genome indicated less methylation in GRIK2 (glutamate receptor, ionotropic kainate 2) and BEGAIN (brain-enriched guanylate kinase-associated protein) associated with depressive psychopathology.^105^ Single-cell epigenomics^106^ is based on sequencing single nuclei, barcoding all the transcripts from each nucleus, and using the expression pattern to sort the nuclei into specific cell subtypes. This technique may be an excellent approach to further investigate changes in specific genes in specific brain cells in MDD-associated DNA methylation; however, single-nucleus, postmortem brain studies of MDD are rare.

## Histone modifications in the pathogenesis of MDD

Histones are essential proteins rich in lysine and arginine residues in eukaryotic somatic chromatin. The DNA molecule (approximately 150 bp) is enveloped by histone octamers, which comprise two sets of fundamental histones (H2A, H2B, H3, and H4), to form a single nucleosome, the basic repeating subunit of chromatin.^107^ Histones regulate gene expression both positively and negatively.^108^ This is mainly governed by posttranslational modifications catalyzed by enzymes that act on particular amino acid residues located on histones. The H3-H4 tetramer is stable and allows histone modifications to be heritable epigenetic marks.^109^ The lengthy tails extending from the nucleosome of H3 and H4 histones can be covalently changed in several locations. Methylation, acetylation, phosphorylation, ubiquitination, SUMOylation, crotonylation, citrullination, and ADP-ribosylation are a few modifications of the tail.^110,111^ Acetylation or methylation of lysine or arginine residues are the most common modifications observed, which alter the interactions between histones or transcription factors and DNA, regulating gene expression.^112^ Furthermore, studies have suggested that the patterns of DNA methyltransferase localization, DNA methylation, and actively transcribed gene bodies may be specified by histone modifications.^113,114^

### Histone acetylation in stress response and neuroplasticity

**Fig. 3:**
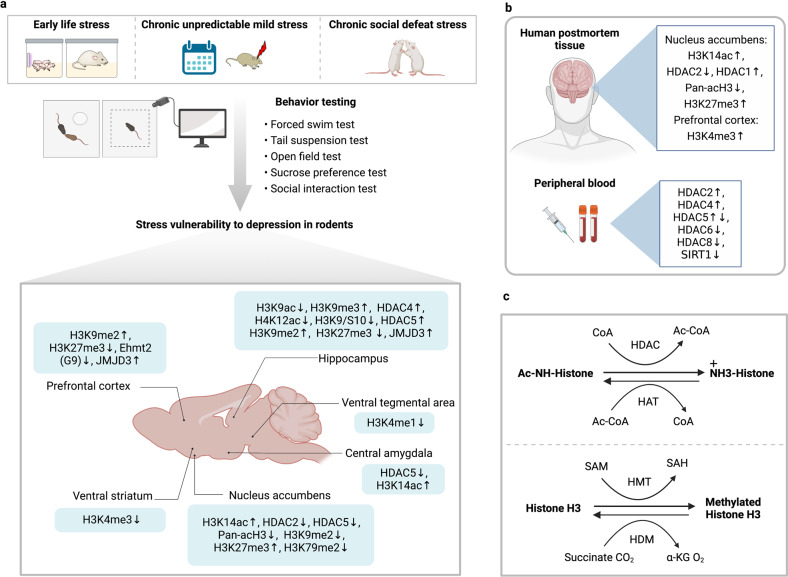
Different types of histone modification changes in different brain regions in stressed animals and depressed humans. **a** Animal models and behavior analyses for studying the relationship between stress vulnerability to epigenetic changes and depression. Recent studies using animal models show brain region-specific histone modification changes. The NAc^104,130,158,167,168,211^ and hippocampus^136,137,148,170,175,513^ are the most studied brain regions for histone modification, with both consistent and conflicting findings across different studies. Different types of histone modification are also observed in other brain regions, such as the prefrontal cortex^175^. **b** Histone modification changes based on human postmortem tissue^130,158,165,168^ and peripheral blood, collected in both cases from depressed individuals^159,160^. **c** Chemical reactions involved in histone acetylation and methylation. NAc nucleus accumbens, CoA crotonyl-coenzyme A, HDAC histone deacetylases, HAT histone acetyl transferases, SAM S-adenosyl methionine, HMT histone methyltransferases, HDM histone demethylases, SAH S-adenosyl homocysteine, α-KG α-ketoglutarate. ↑ Increased changes compared with controls; ↓ decreased changes compared with controls; ↑↓ both increased and decreased changes were reported across different studies

Histone acetylation at lysine residues (mainly including K9, K14, K18, K23, and K27), is commonly linked to the activation of gene transcription. Acetylation is typically observed at the transcriptional start sites and enhancers of genes that are actively transcribed.^115^ The “writers” that mediate histone acetylation include histone acetyltransferases (HATs) for the addition of the acetylation mark. The “eraser” histone deacetylases (HDACs) eliminate acetyl groups from lysine residues (Fig. 3c); as a result, the ionic interaction between histones and DNA increases, DNA packs more tightly and chromatin is more highly condensed. HDACs are grouped into two families: the traditional HDACs and the NAD+-dependent, silent information regulator (SIR2) family of HDACs.^116^ The former includes three phylogenetic classes: I (HDAC1, 2, 3, and 8), II (HDAC4, 5, 6, 7, 9, and 10), and IV (HDAC11). The SIR2 family of HDACs, sometimes called class III HDACs or sirtuin (SIRT), deacetylates both histone and nonhistone proteins, modulating many cellular processes^117,118^, and influencing gene expression.^119^

As a stress-related disorder, major depression is associated with an abnormal stress response system. For example, excessive cortisol release in response to stress and impaired GR-mediated feedback inhibition have been known for decades.^120,121^ The NR3C1 gene encodes for the GR,^122,123^ which is an essential component of the HPA axis. As a transcription factor, the GR interacts with and influences the histone landscape, playing a critical role in shaping it.^124,125^ Animal models for studying depression have been established based on exposure to different forms of stressors in rodents such as early life stress (ELS), chronic social defeat stress, and chronic unpredictable mild stress.^126^ These models, combined with the ability to assess anhedonia, anxiety- and depression-related behavior objectively and learned helplessness in rodents, have helped to elucidate the link between stress and vulnerability to depression as mediated by epigenetic changes (Fig. 3a). Histone acetylation changes, primarily mediated by HDAC, impact the stress response, depression-like behavior, and antidepressant effects.^104,127–129^

#### Class I HDACs involved in antidepressant effects and neuronal plasticity

Early evidence supporting the involvement of HDACs in the stress response and antidepressant action showed that mice exposed to chronic social defeat stress experienced a temporary reduction followed by a lasting escalation in histone acetylation levels (H3K14ac), which was connected to decreased histone deacetylase 2 (HDAC2) levels in the NAc.^130^ Infusion of HDAC inhibitors (i.e., MS-275) into the NAc reversed global gene expression patterns in the NAc and improved depression-like behaviors. The observation that MS-275 had antidepressant effects suggested that histone acetylation plays an adaptive role in the response to stress. This was supported by another study showing that animals overexpressing HDAC2 in the NAc region exhibit more depression-like behavior.^104^

Although primarily manifested as depression, MDD also results in cognitive impairments.^131,132^ Neuronal plasticity and cognitive function are associated with transcriptional changes regulated by HDAC-mediated epigenetic modification.^133^ The overexpression of HDAC2 specifically in neurons resulted in a decrease in dendritic spine density, synapse number, synaptic plasticity, and memory formation in mice. However, prolonged administration of HDAC inhibitors improved synapse number and learning impairment.^134^ Furthermore, a promoter occupancy analysis has revealed a link between HDAC2 and the promoters of genes that play a role in synaptic plasticity and memory formation.^134^ Consistent with these findings, viral-mediated knockdown of HDAC2 can restore both structural and synaptic plasticity, ultimately leading to an improvement in memory loss associated with neurodegeneration.^135^

#### Class II HDACs have opposite roles in different brain regions related to depression

Studies with class II HDACs (i.e., HDAC4 and HDAC5) revealed different roles for these molecules. The AChE gene, which encodes the acetylcholine-hydrolyzing enzyme acetylcholinesterase, was downregulated in the hippocampus after stress and accompanied by decreased acetylation and increased trimethylation of H3K9 at the corresponding promoter, as well as HDAC4 accumulation in hippocampal neurons. These effects were reversed drastically by administration an HDAC inhibitor, and reduced hippocampal HDAC4 levels restored the long-lasting behavioral deficit.^136^ In addition, overexpression of HDAC4 in the hippocampus caused adult rats to be depressive but not anxious.^137^

HDAC4 exhibits high expression levels in the forebrain and is enriched in neurons.^138,139^ It shuttles between the cell nucleus and cytoplasm.^140^ Cytoplasmic localization of HDAC4 is maintained by HDAC phosphorylation, suppressing the binding of the transcription factor MEF2,^141^ whereas HDAC4 dephosphorylation by calcineurin allows nuclear translocation.^142^ Nuclear HDAC4 functions as a transcriptional repressor, downregulating the expression of numerous plasticity-related genes, that may mediate effects on learning and memory impairment in mice.^143,144^ In contrast, the role of cytoplasmic HDACs is less understood. HDAC4 in the cytoplasm may have neuroprotective effects.^145–147^ Inhibiting HDAC4 delayed the formation of huntingtin protein cytoplasmic aggregates, while the levels of BDNF transcripts were restored, resulting in the restoration of neuronal and cortico-striatal synaptic function in mouse models of neurological disorders.^147^

Regarding HDAC5, chronic imipramine (a tricyclic antidepressant) administration to mice with chronic social defeat behavior was linked to a selective downregulation of HDAC5 in the hippocampus; viral-mediated HDAC5 overexpression blocked the antidepressant-like effect of imipramine.^148^ In another study of chronically stressed rats, a significant decrease in histone acetylation (H4K12Ac) and phosphor-acetylation (H3K9/S10) was observed in CA3 and dentate gyrus (DG) in stressed animals compared with control animals, along with increased HDAC5 expression. HDAC5 seems to play a role of promoting depression in the hippocampus, but in other brain regions, HDAC5 may have the opposite effect. For example, mice exposed to chronic social defeat stress showed decreased HDAC5 expression in the NAc, whereas chronic administration of imipramine increased HDAC5 expression in NAc,^149^ suggesting a pro-resilient role of HDAC5 in the NAc. Rats exposed to variable mild stress manifested decreased HDAC5 in the central amygdala (CeA).^150^ Acetylation in the amygdala could potentially be an advantageous adaptation. Transiently increased H3K14 acetylation in the amygdala of mice followed by chronic social defeat stress. Conversely, the injection of an HDAC inhibitor into the amygdala reversed social avoidance.^127^ Changes of HDAC5 in the amygdala resemble the observations made in the NAc of mice following exposure to stress, suggesting a homeostatic role, in contrast to the pro-depressive effect in the hippocampus. The reason why HDAC5 plays opposite roles in different brain areas may lie in its involvement in diverse complexes that target specific gene subsets associated with depression.

#### Class III HDACs associate with depression

In addition to classical HDACs, class III HDAC (i.e., SIRT), which deacetylates histones and nonhistone proteins, also appears to be associated with hippocampal neuroplasticity and depression-like behavior.^151–153^ Activation of SIRT in the hippocampus exerts antidepressant effects and blocks abnormal dendritic structures. Blocking SIRT1 function in the hippocampus increases depression-like behaviors.^151^ SIRT1 is also one of the first genes (SNPs; rs12413112) identified using GWAS to be associated with MDD.^154,155^ Furthermore, lower SIRT expression levels in peripheral blood samples of MDD patients^156^ align with findings from other studies.^157^ Taken together, these findings suggest that activating SIRT1-dependent pathways may be a potential therapeutic strategy for MDD.

#### Differential histone modification in human samples

Some studies examined histone modifications in the human postmortem brain or the expression of histone-modifying enzymes in the peripheral blood cells in MDD (Fig. 3b). Expression of Rac1, involved in synaptic structure regulation, was low in NAc postmortem tissues in MDD and associated with lower histone H3 pan acetylation and more histone H3K27 trimethylation.^158^ In addition, differential brain expression of HDACs was found in the postmortem in MDD.^130,159^ Expression of HDAC2 and HDAC5 was higher during a depressive state, compared with remission, in peripheral white blood cells of patients diagnosed with MDD and bipolar disorder (BPD).^159^ The expression of HDAC6 and HDAC8 was lower regardless of mood states compared with controls in BPD, while the HDAC4 expression was higher only in a depressive state.^159^ This study links altered expression of HDACs with depression and is consistent with a potential homeostatic role of histone acetylation in response to stress and depression. Antidepressant treatment caused higher peripheral HDAC5 expression to decrease to control levels after about eight weeks of treatment.^160^ It is impossible to separate in clinical studies whether biology that fluctuates with the severity of illness is a homeostatic response, a measure of the stress due to the illness severity, or part of the pathophysiology responsible for illness severity.

Collectively, stress-induced depression is associated with decreased histone acetylation. HDACs appear to be involved in neuroplasticity, neuronal survival, and cognition and may become potential targets of antidepressant intervention. HDAC inhibitors restore memory deficits in mice,^161^ but it remains to be seen whether modifying HDACs ameliorates cognitive impairments and depression in humans.

### Histone methylation in stress response and depression

Histone methylation involves the transfer of methyl groups to amino acids in histone proteins,^162^ occurring at various locations along the histone tails, resulting in the addition of one, two, or three methyl groups to the lysine or arginine residues. In contrast to histone acetylation, histone methylation is in general linked to transcriptional repression. However, in some cases, the relationship between histone methylation and transcription depends on the level of methylation and the site of the residue. For example, methylation of H3K9 and H3K27 leads to repressed gene expression;^163^ while histone methylation of H3K4 in the promoter regions results in relaxed chromatin, which promotes gene expression.^164^ The enzymes that facilitate histone methylation include histone methyltransferases (HMTs) and histone demethylases (HDMs), which transfer or remove methyl groups from target residues using S-Adenosyl methionine as a methyl donor.

Trimethylation of histone 3 lysine 4 (H3K4me3) is one of the most characteristic histone modifications. When exposed to chronic unpredictable mild stress (CUMS), the level of H3K4me3 was decreased at the promoter region of the Gdnf gene, which led to altered Gdnf expression in the ventral striatum in mice.^104^ Interestingly, enhancement of H3K4me3 was found in postmortem brain tissue (BA10) in MDD^165^: the enriched H3K4me3 was found at transcriptional start sites of synapsin 1, relevant to increased expression of synapsin 1a and synapsin 1b. These synapsins are critical in synapse function and plasticity.^166^ Other forms of histone methylation include H3K9me2 and H3K27me3, are repressive in the gene promoter region in response to stress.^167,168^ Rats exposed to the early stress of maternal separation (ES) exhibited decreased H3K9me2 modification at the BDNF IV promoter site, along with increased BDNF levels, enhanced hippocampal neurogenesis, and better cognitive performance in both the postnatal life and young adulthood. Interestingly, middle-aged rats that experienced early maternal separation exhibited impaired cognition, reduced H3K9me2 regulation of the BDNF expression, and opposite changes in the hippocampal neurogenesis, suggesting both biphasic and distinct, age-dependent changes in the histone methylation in response to ES.^169^ Another study showed elevated H3K9me2 levels and reduced BDNF levels in the hippocampus and medial prefrontal cortex (mPFC) in CUMS rats showing depression-like behaviors.^170^ In addition, higher H3K9me2 levels at the calmodulin-dependent protein kinase II α (CaMKIIα) promoter and inhibition of CaMKIIα were found in MDD and mice following antidepressant administration.^171^ The activation of H3K27me3 in the NAc of mice exposed to social defeat stress inhibits the expression of RAS-related C3 botulinum toxin substrate 1 (Rac1), a Rho GTPase–related gene known for synaptic structure regulation.^158^

In parallel to prominent findings for HDACs, related enzymes for histone methylation and demethylation (HMTs and HDMs) also were potentially crucial in depression. Higher expression of Setdb1, an H3K9-specific HMT in the mouse forebrain, was associated with changes in the composition of NMDA receptor subunits, as well as other molecular modifications resulting from suppressive chromatin remodeling at specific target genes.^172^ This regulation led to anhedonia, despair, and learned helplessness in behavioral paradigms. In line with these findings, downregulation of HMT Ehmt2 (G9a) was associated with loss of repressive histone methylation (H3K9meX) after chronic stress.^173^ Protein arginine methyltransferase 1 (PRMT1), another HMT, when knocked out, improved depression-like behavior, along with upregulated expression of BDNF and postsynaptic density protein 95 (PSD95).^174^ The histone lysine demethylase jumonji domain-containing 3 was also upregulated in the prefrontal cortex and hippocampus of rats exposed to CUMS.^175^

### Other types of histone modifications affecting depression

Several uncommon histone modifications, such as histone crotonylation, histone phosphorylation, and histone β-hydroxybutyrylation, are shown to be involved in the pathophysiology of MDD.^176–178^ Histone crotonylation was previously shown to occur at the promoters or enhancers of gens that are actively transcribed, and play a role in spermatogenesis.^111,179^ Similar to histone acetylation, histone crotonylation is catalyzed by HATs, which add a crotonyl group from crotonyl-coenzyme A (CoA) to amino acid residues of histones,^111^ while HDACs 1/2/3 and sirtuin 1/2/3, act as decrotonylases.^180–182^ In the medial prefrontal cortex of mice vulnerable to chronic social defeat stress, histone crotonylation was inhibited. In contrast, chromodomain Y-like protein (CDYL), which acts as both a crotonyl-CoA hydratase and a histone methyllysine reader, was selectively upregulated.^183^

Histone phosphorylation mainly occurs on serine and threonine residues, though some studies have found that tyrosine residues also can be phosphorylated.^184^ Histone phosphorylation in the central nervous system is involved in stress response. For example, the expression of H3S10 phosphorylation (H3S10p) was increased in the hippocampus in mice exposed to fear conditioning.^185^ Rats exposed to novelty stress also showed an induction of H3S10p at the c-fos promoter site in the dentate gyrus.^186^ To date, few studies have investigated the relationship between histone phosphorylation and MDD. Rats exposed to forced swimming stress showed an increase in H3 phosphorylation in the infralimbic (ILCx) and prelimbic area (PrLCx) of the prefrontal cortex.^187^

In 2006, histone β-hydroxybutyrylation was reported as a newly-histone modification. It is dynamically controlled by the concentration of hydroxybutyrate in cells.^177^ However, the mechanism by which a β-hydroxybutyryl group is added to histones remains unknown.^177,188^ It has been demonstrated that β-hydroxybutyrate exerts an antidepressant-like effect in a rodent model of chronic unpredictable stress^189,190^. Injection of β-hydroxybutyrate improved depression symptoms and reversed the reduction of H3K9 β-hydroxybutyrylation and increased BDNF expression. This study emphasized that metabolite changes after stress contribute to histone modification.^191^ However, further research is necessary to investigate the homeostasis of other types of histone modifications and the pathophysiological changes associated with MDD.

### Susceptibility and adaptation to stress mediated by histone modification

When exposed to stressful events, only a subgroup of individuals develops major depression.^192–195^ Two genetically diverse mouse strains show how epigenetic changes underlie susceptibility and adaptation to chronic stress during adulthood.^104^ The interplay of genetic factors and environmental stressors can be mediated by histone modifications and DNA methylation in the ventral striatum, thus contributing to behavioral responses to stress.^104^ Specific cell types and epigenetic changes affect stress susceptibility in an opposing manner.^196^ *Fosb*-targeted histone acetylation or methylation in different types of medium spiny neurons (i.e., D2-type vs. D1-type) controls stress susceptibility and resilience to social stress.

Experiencing stress in the early life, commonly known as Early life stress (ELS), increases the likelihood of developing depression, suicidal behavior, and various other psychiatric disorders during adulthood.^26,197–200^ Depressive patients reporting childhood trauma show earlier onset, chronic course, and poorer response to antidepressant treatment.^201^ In animal models, long-lasting effects of ELS have been documented.^202–207^ ELS increased the vulnerability to adult stress in rodents^208^. ELS may not cause immediate behavioral abnormalities but instead causes long-lasting transcriptional alterations that prime critical brain regions to be hyper-responsive to later stress and prone to develop a depression-like state.^209,210^ This was illustrated by a “two-hit” stress model in mice wherein ELS (stress hit number 1) increased susceptibility to depression-like behavior in response to social defeat stress (stress hit number 2) during adulthood.^209^

Enduring transcriptional alterations were observed in the ventral tegmental area (VTA) in ELS, mediated by the developmental transcription factor orthodenticle homeobox 2 (OTX2). Furthermore, histone methylation (H3K4me1) changes were found in genes targeted by OTX2 binding. The same stress model^211^ revealed long-lasting histone modifications in the NAc after ELS. ELS-induced depression susceptibility was caused by decreased histone methylation (H3K79me2) and induction of the DOT1L and KDM2B enzymes that regulate this histone methylation in D2-type medium spiny neurons. In addition, systemic delivery of DOT1L inhibitor reversed ELS-induced behavioral deficits without detectable side effects, suggesting a potential therapeutic target.^211^ The regulation of epigenetic markers is complex, involving upregulation or downregulation at hundreds of loci on a genome-wide scale. Thus, more studies are needed using genome-wide histone code profiling such as chromatin immunoprecipitation followed by sequencing.^212^

## Noncoding RNAs participate in the development and treatment of MDD

**Fig. 4:**
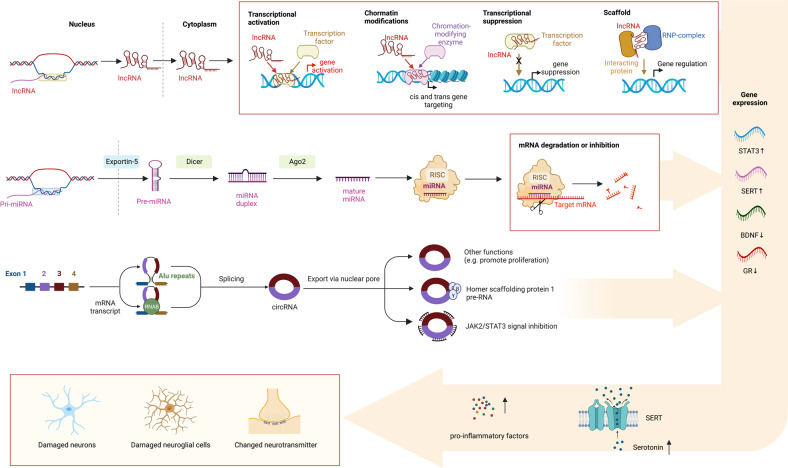
Noncoding RNA in depression: generation, mechanisms of function, and effects. DNA constitutes an essential part of the human genome and contributes to the formation of noncoding RNA after transcription. For lncRNAs, multiple mechanisms are involved in the pathophysiology of depression: transcriptional activation: lncRNAs can activate the expression of target genes via (1) recruiting transcriptional factors to upstream open reading frames; (2) suppressing transcriptional factors to upstream open reading frames; (3) recruiting chromatin modifying factors to alter chromatin structure; (4) suppressing interacting proteins and RNP-complexes to target genes. For circRNAs, they are generated from back-splicing and canonical splicing of an mRNA transcript. These circRNAs are associated with homer scaffolding protein 1, regulate cell proliferation, and inhibit JAK2/STAT3 signal, leading to neuropathological changes related to depression. For miRNAs, multiple mechanisms, including mRNA degradation or inhibition by the RISC complex, activate depression. These mechanisms induce gene expression changes, which are associated with several molecular pathways of depression. RISC RNA-induced silencing complex, RNP ribonucleoprotein complexes, STAT3 signal transducer and activator of transcription 3, SERT serotonin transporter, BDNF brain-derived neurotrophic factor, GR glucocorticoid receptor, JAK2-STAT3 Janus kinase 2 and signal transducer and activator of transcription 3

MicroRNAs (miRNAs), circular RNAs (lncRNAs), long noncoding RNAs (lncRNAs), and other as yet unidentified RNAs are referred as noncoding RNAs (ncRNAs), which do not code for peptides or proteins.^213,214^ Although ncRNAs cannot encode proteins, they can influence the expression of other genes through multiple mechanisms to cause a wide range of disorders.^215–218^ ncRNAs can regulate expression of genes via the translation or transcription of mRNAs, the methylation of DNA and RNA, or as a modular scaffold of histone modification complexes.^219^ Studies have demonstrated that significantly altered ncRNAs expression in MDD relative to healthy subjects. At the same time, antidepressants can also alter the abnormal expression of ncRNAs.^220^ ncRNAs may influence the pathophysiology of depression by regulating neuronal function, neurotransmitter release, and microglia (Fig. 4).^213,221,222^

### miRNAs and the development of depression

**Fig. 5:**
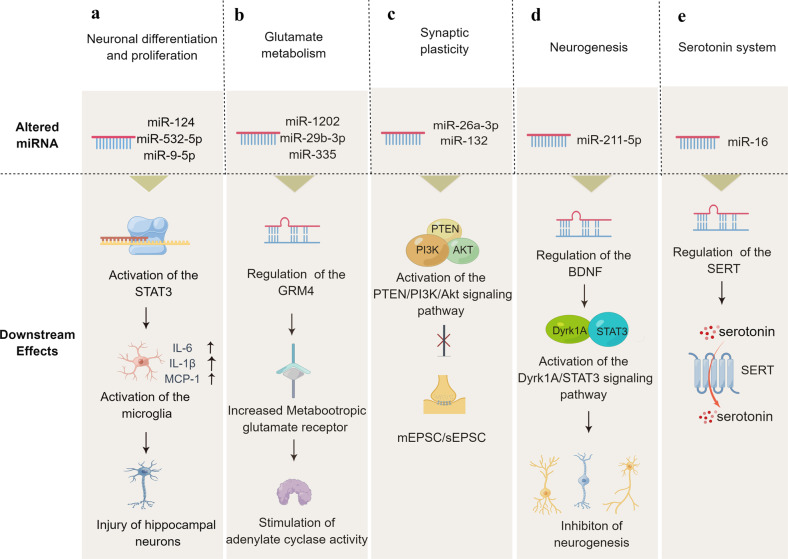
Functional mechanisms of representative miRNAs involved in depression. A variety of miRNAs can influence neurodevelopment, synaptic plasticity and neurotransmitters by regulating their target genes, thus leading to depression. PTEN phosphatase and tensin homolog deleted on chromosome ten, PI3K phosphoinositide 3-kinase, GRM metabotropic glutamate receptor, mEPSC/sEPSC miniature excitatory postsynaptic current/spontaneous excitatory postsynaptic current, SERT serotonin transporter, BDNF brain-derived neurotrophic factor

MicroRNA molecules are highly conserved small (21–24 nucleotides) RNA molecules.^223^ Over 60% of human protein-coding genes are dynamically regulated by miRNAs, thereby influencing cell growth and differentiation.^224^ The mechanistic details of miRNA synthesis and maturation have been reviewed^225^ and are summarized here. RNA polymerase II converts an intragenic or intergenic miRNA gene into a pri-miRNA (Fig. 5).^226^ Following several cleavage and maturation steps, the pre-miRNA is exported to the cytoplasm and incorporated with RNA-induced silencing complexes (RISCs).^227,228^ miRNA sequences guide the RISC to target mRNA transcripts, resulting in translational cleavage or repression of mRNA.^226^ Traditionally it was thought that the miRNAs targeted the 3′ UTRs of transcripts but it has become apparent that 5′ UTRs, coding sequences, and promoters also can be targeted for repression.^229^ Because miRNAs have imperfect sequence recognition, they may bind to multiple target genes and modulate their expression.^226^ In contrast, aberrant miRNA can contribute to cancers, cardiovascular diseases, metabolic diseases, immune-mediated disorders, and neurological disorders, etc.^230,231^ For example, miR-106b-5p is an oncogenic miRNA that appears to be upregulated in different cancers, such as hepatic cancer,^232^ cervical cancer,^233^ gliomas,^234^ and gastric cancer.^235^ Likewise, miR-34 dysregulation is related to different psychiatric disorders, including schizophrenia^236^, Alzheimer’s disease,^237^ and MDD.^238^

miRNAs have been evaluated in MDD by examining their level in peripheral blood, cerebrospinal fluid, and postmortem cortex of depressive patients and in animal models of depression.^239–241^ The depression susceptibility is highly associated with the miRNA polymorphisms.^242^ In peripheral blood of depressed patients, multiple miRNAs, such as miR-330-3p, miR-345-3p, miR-425-3p, and miR-24-3p, were reportedly altered.^243^ In the prefrontal cortex (BA9) of MDD patients, other 21 miRNAs, including miR-142-5p, miR-101, miR-137, and miR-301a, was downregulated.^244^ MiRNA expression levels were also found to be altered in BA10,^244^ BA44,^245^ anterior cingulate cortex (ACC),^246^ and other brain regions in MDD.^247^ The link between miRNAs and the pathophysiology of depression^248–252^ may be implicated by the fact that miRNAs regulate neuronal regulation, neurotransmitter regulation, and microglia activation.^253,254^

#### Neuronal regulation by miRNAs

In depressed patients, fewer pyramidal neurons are found in the hippocampus and prefrontal cortex.^255,256^ Cell loss, neuronal atrophy, and alterations in synapse density are also observed in depression animal models.^257^ MiRNAs may mediate these effects in depression by influencing neuronal production, differentiation, proliferation and synaptic plasticity.^258^

As shown in Fig. 5, the expression of miR-124, miR-532-5p, and miR-9-5p alters in tissues from depression patients/animal models, activating signal transducer and activator of transcription 3 (STAT3) and mediating the disruption of neuronal differentiation and proliferation in depression.^259–262^ Similarly, miR-26a-3p^263^ and miR-132^264^ can induce depressive symptoms by activating the phosphatase and tensin homolog (PTEN)/phosphoinositide 3-kinase (PI3K)/Akt signaling pathway, which is one of the main pathways for regulating synaptic plasticity. Low hippocampal miR-211-5p can inhibit neuronal neurogenesis and induce depression-like behaviors via the promotion of the Dyrk1A/STAT3 signaling pathway.^265^ Low miR-218 expression may influence synapse formation and synaptic plasticity of the mPFC by repressing the gene expression of Netrin-1 guidance cue receptor gene *DCC* in mouse models of social defeat stress.^266^ These data demonstrate the crucial role of miRNAs in neuronal differentiation, production, and maintenance.

#### miRNAs regulate neuroglial cells

The peripheral and central nervous systems (CNS) both contain neuroglial cells, such as microglia, astrocytes, and oligodendrocytes. The neuroglial cells serve as the first line of defense for the brain, not only by recognizing pathogens and repairing damaged tissue but also by secreting neurotrophic factors and producing chemokines and cytokines.^267^ Chronic unpredictable stress (CUS) model mice showed changes in neuroglial numbers and morphologies in the hippocampus. Postmortem studies of MDD cases show deficits in neuroglial numbers in the neocortex.^268^ Therefore, the occurrence and development of depression may be associated with neuroglial cells.^269^

The protein WNT2 plays pivotal role in embryonic development, the adult hippocampal neurogenesis, and development of the nervous system. In depression, more miR-199a-5p was noted in microglia suppressing WNT2 expression through the CREB/BDNF signaling regulation.^270^ The downregulation of miR-124 in depression also promotes the activation of BV2 microglia by activating STAT3.^259^ This increases inducible nitric oxide synthase and proinflammatory cytokine (MCP-1, IL-6, TNF-α, and IL-1β) secreted by BV2 cells, which induces depressive symptoms. In addition, miR-146a-5p is transferred by microglia to the hippocampus DG, where it targets Krüppel-like factor 4 (KLF4) and inhibits neurogenesis and the spontaneous discharge of excitatory neurons.^254^

#### Regulation of neurotransmitters by miRNAs

Studies have shown that miRNA-mediated disturbance of neurotransmitters, including glutamate, dopamine, serotonin and norepinephrine, can lead to neuronal damage, and loss of trophic effects and thereby potentially contribute to the pathogenesis of depression.^271–274^ Glutamate and its receptors are associated with many neuropsychiatric illnesses, including depression.^275,276^ MiR-335, miR-1202, miR-29b-3p, and miR-134-5p may regulate depression through inhibiting the expression of *GRM4* gene (Fig. 5), that in turn affect the influx of Ca^2+^ into the prefrontal cortex neurons and the extracellular concentration of glutamate.^277–283^ The monoamine oxidase A (MAO-A) enzyme modulates monoamine neurotransmitter levels. miR-142, miR-34a, and miR-34c indirectly regulate MAO-A, potentially impacting monoamine neurotransmitters.^284^ In addition, miR-16 upregulation in depression inhibits the translation of the serotonin transporter (SERT) gene, affecting serotonin function in the hippocampus.^285,286^

### circRNAs engage in the development of MDD

circRNAs are covalently closed circular RNA molecules that typically contain exons, formed by direct splicing 3′ and 5′ terminal ends generated from precursor mRNA.^220,287^ These RNA molecules function as a class of post-transcriptional regulators.^288^ Typically, circRNAs regulate the expression levels of downstream genes at several levels, including mRNA transcription, splicing, and translation^287^. In addition, it has been reported that some circRNAs can translate to peptides.^289^ Several studies have demonstrated that circRNAs are abundantly expressed in the brain. And also, circRNAs are involved in the pathogenesis of neuropsychiatric disorders, impacting neuron development, cognitive function, and synaptic plasticity^290–292^.

#### Neuronal regulation by circRNAs

CircRNAs involve in regulating central neural system development. They are highly expressed in the brain from the embryonic stage to adulthood and substantially upregulated during the differentiation and maturation of neurons.^293^ Many circRNAs are significantly upregulated at different time points of neuron maturation in vitro.^293^ In vitro studies have shown that overexpression of circPTK2 can induce neuronal apoptosis via miR-29b inhibition, preventing inflammatory response and protecting against neuronal apoptosis.^294^ Conversely, circPTK2 silencing inhibited JAK2/STAT3 and decreased IL-1β, thereby ensuring neuronal survival.^294^

#### circRNAs regulate neuroglial cells

It was reported that cirRNAs regulate neuroglial activities. For example, one study revealed that the gut microbiota-circHIPK2-astrocyte axis was involved in the development of depression in a stress mouse model.^295^ Fecal microbiota transplantation significantly alleviated astrocyte dysfunction and depression-like behaviors in recipient depressive mice via inhibition of circHIPK2 expression.^295^ Similarly, another study demonstrated that overexpression of circSTAG1 significantly ameliorated depression-like behaviors and astrocyte dysfunction in the stress mice model.^296^ In addition, a recent study using two different MDD mouse models demonstrated the presence of a close relationship between depression and decreased circDYM and revealed that increased circDYM expression could significantly attenuate depressive-like behavior and inhibit microglial activity.^290^

#### Regulation of neurotransmitters and synaptic plasticity by circRNAs

circRNAs were also observed to be linked to neurotransmitter function and synaptic plasticity and have a higher expression in the neuropil area compared to their mRNA isoforms.^297^ Studies have shown many host genes of circRNA were enriched in the pathway of synaptic activities and neurotransmitter secretion.^289,297,298^ For instance, circHomer1 derived from the homer scaffolding protein 1 pre-RNA can regulate some synaptic structure during the development and neuronal plasticity.^297^ Furthermore, a study using a murine cell line model of Huntington’s disease identified 23 differentially expressed circRNAs significantly enriched in the MAPK, dopaminergic synapse, and long-term depression pathways.^299^

### lncRNAs participate in the process of depression

lncRNAs are more than 200 nucleotides in length and cannot encode proteins, but they share some characteristics with messenger RNAs.^300^ lncRNAs, for instance, are transcribed by RNA polymerase II; they are 5′ capped with a polyA tail and have multiple exons. Because of their lack of conservation across species and lower expression levels than mRNAs, lncRNAs were initially regarded as transcriptional noise or junk.^301^ However, some long noncoding RNAs are involved in regulating gene expression, including transcriptional regulation, RNA regulation, chromatin modification, and posttranscriptional regulation of protein activity and localization.^302^

Highly expression of lncRNAs in the brain is crucial for maintaining neural stem cells, neurogenesis, neural plasticity and cognitive function, etc.^303^ For example, brain cytoplasmic lncRNA (BC1), one of the earliest lncRNAs studied in the brain, regulates metabotropic receptor signaling.^304^ In addition, neurogenesis-associated lncRNAs may act as guides for proteins associated with neurogenesis, including REST, SOX2 and SUZ12. Furthermore, RNAi knockdown of some lncRNAs impairs neuronal differentiation, suggesting that lncRNAs play critical roles in neurogenesis.^305^ Several recent studies have indicated that lncRNAs are crucial in the development of numerous neurological disorders, including autism, schizophrenia, and depression.^306–309^ However, further research is needed to determine their signal pathway targets and regulation.

Levels of 60% (217 out of 364) of the 364 lncRNAs expressed in the rostral cingulate cortex of normal individuals differed from those in MDD.^310^ Using whole-genome sequencing, a recent study found that lncRNAs accounted for 30% of the differences between patients with depression and control populations.^311^ In MDD patients, four lncRNAs (PCAT1, MER11C, Y5, and PCAT29) levels were higher, and one lncRNA (RMRP) was lower in peripheral blood leukocytes.^312^

#### Regulation of neuronal cells by lncRNAs

In MDD patients, LncRNA TCONS 00019174 levels were lower, and phosphorylated-GSK3 and -catenin was higher in the hippocampus, potentially causing neuronal damage.^313^ Furthermore, viral-mediated overexpression of lncRNA TCONS 00019174 aggravated depression status in depressed mice model, indicating that lncRNA TCONS 00019174 may be linked to the pathogenesis of depression.^313^ Differentially expressed lncRNAs showed correlations with 18 synapse-related functions, which showed that lncRNA-directed regulatory machinery might mediate synaptic dysfunction in depression.^314^

#### Regulation of neuroglial cell by lncRNAs

Administration of lncRNAs can treat depression at the neuroglial cell level, which promotes the pathogenesis of depression. More M1 microglia were found in the hippocampus of depressed rats and fewer M2 microglia, as well as a negative regulation factor of the lncRNA uc.80.^315^ Elevated expression of lncRNA uc.80 can reduce depression-like behaviors in rats and hippocampal neuron apoptosis in vitro and in vivo.^315^ Consequently, lncRNA uc.80 may be a potential target of treatment for depression.

#### Neurotransmitters regulated by lncRNAs

Researchers have shown that upregulated lncRNA NONHSAG045500 inhibits the expression of the 5-HT transporter SERT which influences 5-HT transmission in depression.^316^ Meanwhile, the concentration of 5-HT was increased by interference with the levels of NONHSAG045500. NONHSAG045500 may modify the transport of monoamine neurotransmitters.^316^

### Effective antidepressant treatment is associated with ncRNA alteration

There are approximately six major classes of antidepressants used worldwide. Classical antidepressants include the noradrenaline reuptake inhibitors (NRIs), selective serotonin reuptake inhibitors (SSRIs), combination serotonin and norepinephrine reuptake inhibitors (SNRIs), and older drugs like tricyclic antidepressants and monoamine oxidase inhibitors.^317,318^ New-generation antipsychotics and drugs targeting the alpha2-adrenergic autoreceptor like mirtazapine are also used.^319^ In 2019, the Food and Drug Administration in the United States approved Ketamine, a newer medication for patients with depression and suicidal tendencies not responding to standard antidepressants. It is reported that almost 60% of patients fail to recover after undergoing one antidepressant trial,^320–322^ and misleading reviews have ignored a vast body of efficacy data and even questioned whether antidepressants work at all.^323,324^ Accordingly, a better understanding of how antidepressants work and why they do not work in some patients is needed. Some evidence suggests that SSRIs, SNRIs, or ketamine may exert their influence by affecting miRNAs.^249,325–328^

#### Noncoding RNA modulation in response to SSRIs

Application of escitalopram in depression of patients altered the levels of 30 miRNAs,^329^ have triggered studies finding that miRNAs may be downstream regulators mediating the antidepressant effects of SSRIs.

Fluoxetine upregulated the expression of miR-16 in the raphe nucleus of mouse,^330^ and downregulated miR-598-5p and miR-451 in hippocampus of mouse brain.^331^ In vitro, fluoxetine upregulated miR-320a, miR-663a, miR-572, and miR-489 in both SK-N-SH and SHSY5Y cell lines.^332^ In addition, fluoxetine can regulate hippocampal lncRNA levels to improve CUMS-induced depression symptomatology.^333^ Among them, lncRNA Gm26917 was significantly upregulated. Paroxetine upregulated the expression of miRNA-451a and downregulated the expression of miRNA-221-3p and miRNA-34a-5p.^334^ A relationship is reported between clinical symptom changes and miRNA expression levels.^334^ Higher miR-1202 levels in MDD patients are reported following eight weeks of citalopram, but no differences in controls.^281^ Plasma miR-132 levels MDD were reduced after two months of citalopram and plasma miR-124 levels increased.^248^ Citalopram increased miRNA-16 in mouse brains and decreased SERT protein levels, while exerting antidepressant effects.^330^ 30 miRNAs were expressed differently in the blood of MDD patients following three months of escitalopram therapy.^329^ Escitalopram administration for four weeks normalized abnormal levels of miR-326 in the NAc of depressed rats, indicating that miR-326 may be a novel target of escitalopram.^335^ The problem with most studies is that they cannot distinguish between miRNA normalization as part of the antidepressant action or miRNA normalization as a consequence of clinical improvement in the patient.

#### Noncoding RNA modulation in response to SNRIs

It has been suggested that SNRIs have better therapeutic efficacy and higher tolerability compared to commonly used antidepressants since of their impact on both norepinephrine (NE) and 5-HT.^336–338^ One of the SNRIs widely used in clinical settings is duloxetine.^339^ Downregulation of miR-3074-5p, miR-146a-5p, miR-24-3p, miR-425-3p, and miR-146b-5p expression in the peripheral blood of depression patients after duloxetine treatment was found.^340^ Administration of duloxetine to rats undergoing CUMS upregulated miR-18a and miR-132 in hippocampus, and downregulated levels of miR-124a and miR-134.^341^ Desvenlafaxine reduced miR-1202 levels in depressed individuals after two months of treatment.^342^ Furthermore, miR-1202 levels in peripheral blood were associated with alterations in brain activity and connectivity in several brain regions.^343^ Perhaps miR-1202 levels may mediate some of the desvenlafaxine’s antidepressant effect through brain circuitry function, possibly via the glutamatergic system.^342^

#### Noncoding RNA modulation in response to ketamine for depression

Ketamine inhibits the N-methyl-D-aspartate (NMDA) receptor, enhances mushroom spine growth in 1–2 days via the mTOR pathway and is known to reduce depression symptoms quickly and robustly.^343^ Ketamine downregulated 18 miRNAs levels and upregulated 22 miRNAs levels in rats.^344^ MiR-206 suppressed ketamine’s ability to up-graduate BDNF expression and is downregulated by ketamine.^344^ Thus, miRNA-206 may moderate ketamine’s antidepressant effect. Ketamine can also increase the expression of miR-29b-3p and result in lower levels of metabotropic glutamate receptor 4 (GRM4) in the prefrontal cortex of depressed rat model, suggesting that the lncRNA Gm26917/miR29b-3p/GRM4 pathway may plays a critical role in both fluoxetine and ketamine therapies in CUMS rats.^345^ Understanding how lncRNA regulation is related to antidepressant treatment effects has become increasingly important to find potential therapeutic targets to improve the treatment of MDD.

## RNA modifications participate in the molecular mechanisms underpinning MDD

**Fig. 6:**
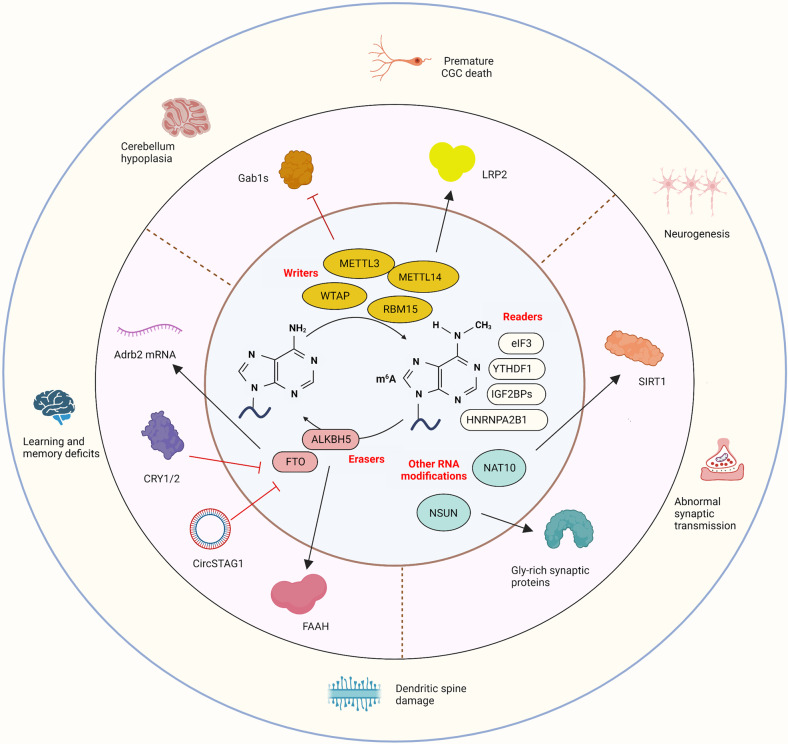
Schematic diagram illustrating how the molecular basis of m^6^A and other reported RNA modifications relates to depression. The modification of m^6^A in MDD is regulated by the action of a complex network of proteins: “writers”, which include METTL3, METTL14, WTAP and RBM15–methylate RNA; “erasers”, which include ALKBH5 and FTO–demethylate RNA; and “readers”, which include eIF3, YTHDF1, IGF2BPs and HNRNPA2B1-recognize m^6^A. Other RNA modifications associated with MDD include N4-acetylcytidine (ac4C) and 5-methylcytosine (m5C). NAT10 can regulate mRNA translation efficiency by catalyzing ac4C while NSUN catalyzes m5C. The molecular consequences of these enzymes involve a variety of pathophysiological mechanisms related to MDD. METTL3 methyltransferase-like 3, METTL14 methyltransferase-like 14, WTAP Wilms tumor 1-associated protein, RBM15 RNA-binding motif protein 15, FTO obesity-associated protein, ALKBH5 Alkb homolog 5, eIF3 eukaryotic initiation factor 3, YTHDF1 YT521-B homology N6-Methyladenosine RNA Binding Protein 1, IGF2BPs insulin-like growth factor 2 mRNA binding proteins, CGC cerebellar granule cells, SIRT sirtuin, Adrb2 Adrenoceptor 2, CRY1/2 circadian regulator cryptochrome 1 and 2, FAAH fatty acid amide hydrolase, LRP2 lipoprotein receptor-related protein 2, Gab1s growth factor receptor-bound protein 2 associated binding protein 1

There are over 100 types of RNA modifications.^346^ Due to their ubiquitous role in cell biology, RNA modifications have been associated with the development of various illnesses, such as cancer, neurological and developmental disorders, and metabolic diseases.^347^ To date, among all RNA modifications, N6-methyladenosine (m^6^A) accounts for two-thirds of all RNA modifications that can be “written”, “read”, and “erased” via the actions of a complex network of proteins^348,349^ and has been widely studied since it was discovered and proposed by Desrosiers in 1975.^350^ M^6^A is a methylation modification of the N at the sixth position of adenosine, which exists not only in mRNA, but also in tRNA, rRNA, and lncRNA.^351^ m^6^A interacts with different reading proteins and related complexes to broadly influence gene expression at multiple levels. There are also many m^6^A modification sites on the mRNA of some proteins that regulate histone modification. Therefore, inhibition of the m^6^A-regulating enzymes may result in increased or decreased histone modification levels.^352^ Here, we review prospective evidence concerning the involvement of RNA modifications, especially m^6^A, in the pathogenesis of depression (Fig. 6), drawing on studies from different species and multiple experimental designs. We discuss the biological effects of RNA modifications in pharmacological and nonpharmacological antidepressant therapies.

### Preclinical and clinical research on m^6^A modification in depression

#### Writers

Scientists have previously shown that the consensus recognition sites for methyltransferase complexes are highly conserved in most eukaryotes.^353^ Core m^6^A methyltransferase complex components include methyltransferase-like 14 (METTL14), Wilms tumor 1-associated protein (WTAP), methyltransferase-like 3 (METTL3), and RNA-binding motif protein 15 (RBM15).^354^ The complex employs S-adenosine methionine (SAM) as a methyl donor to catalyze the formation of methyl groups on the sixth nitrogen element of adenine present in RNA. As the earliest identified component of m^6^A methyltransferase-possessing methylation catalysts, METTL3 plays a role in neural development, and its absence causes hypoplasia of the cerebellum in mice, resulting in ataxia.^355^ METTL3 catalyzes the m^6^A modification of the low-density lipoprotein receptor-related protein 2 (LRP2) mRNA to improve its stability and efficiency in translation. This process relies on reader protein YTH domain containing 2 (Ythdc2), thereby facilitating neurogenesis.^356^ A deficit of METTL3 in mice lessens hippocampal neurogenesis, which induces spatial memory decline, and depression-like behaviors. Targeting the METTL3-Ythdc2-LRP2 axis to regulate neurogenesis may be a promising antidepressant strategy.^356^ Evidence from other studies suggests that upregulated METTL3 can aggravate cognitive impairment in rats exposed to CUMS by mediating m^6^A modification to promote the processing and maturation of pri-miR-221. In addition, this upregulation of METTL3 could increase miR221-3p levels, leading to growth factor receptor-bound protein 2 associated binding protein 1 (Gab1s) inhibition.^357^

#### Erasers

RNA modifications are removed by demethylases, allowing dynamic adjustment. The m^6^A erasers associated with mental stress disorders primarily consist of fat mass and obesity-associated protein (FTO) and Alkb homolog 5 (ALKBH5). The FTO variant rs9939609 in human trials was associated with a lower risk of developing MDD.^358^ A correlation is reported between FTO variants and the severity of MDD.^359^ Lack of the FTO gene impacts anxiety-depression-like behavior in mice.^360^ Adrenoceptor 2 (Adrb2) mRNA appears to be a target of FTO. FTO expression is also related to cognition. Activation of circadian regulator cryptochrome 1 and 2 to inhibit FTO transcription, which in turn inhibits the expression of TrκB in the hippocampus through m^6^A-dependent posttranscriptional regulation, resulted in impaired cognition in mice.^361^ Anxiety-like and depression-like behavior induced by peripheral nerve injury could be reversed in mice by blocking FTO downregulation in the anterior cingulate cortex.^362^ The m^6^A demethylase gene ALKBH5 variant rs12936694 showed an allelic association and genotypic association with MDD.^360,363^ Huang et al. reported that circular RNA STAG (circSTAG1) overexpression hindered the translocation of ALKBH5 into the nucleus, leading to elevated m^6^A methylation of fatty acid amide hydrolase (FAAH) mRNA and degradation of FAAH in astrocytes followed by reduced depression-like behavior and reduced astrocyte loss.^296^

#### Readers

Readers can identify modification signals that react to RNA output, splicing, translation, and degradation by preventing the writer or eraser from adding the modified base, or by recruiting other RNA-binding proteins to facilitate the chemical modification of the RNA. The heterogeneous nuclear ribonucleoprotein (HNRNP) protein family, YT521-B homology domain family (YTHDF), insulin-like growth factor 2 mRNA binding proteins (IGF2BPs) and eukaryotic initiation factor 3 (eIF3) are all members of the “reader” subfamily of m^6^A modification enzymes. According to a previous study, the HNRNP proteins HNRNPA2B1 and HNRNPC exhibit selective binding to mRNAs containing m^6^A.^364,365^ HNRNPA2B1 may be one of eight genes associated with postpartum depression and may have diagnostic value.^366^ By accelerating the translation of specific transcripts in the adult mouse hippocampus, YTHDF1 enhances learning and memory of neuronal stimuli, whereas its absence affects synaptic transmission and long-term enhancement in the hippocampus, leading to deficiencies in learning and memory.^367^

### RNA modifications other than m^6^A are also involved in depression

RNA modifications other than m^6^A have been poorly studied in the field of depression with scant coverage of modifications such as N4-acetylcytidine (ac4C) and 5-methylcytosine (m5C). N-acetyltransferase 10 (NAT10) is a member of the general control non-repressible 5 (GCN5)-related N-acetyltransferase (GNAT) family involved in epigenetic events.^368^ It can regulate mRNA translation efficiency by catalyzing the addition of ac4C to the N4 position on thymidines. In a study conducted on mice, chronic mild stress (CMS) was used to trigger anxiety and depression-like behavior, and the results indicated an increase in NAT10 expression in the hippocampus following the administration of CMS. Pharmacological methods prevented NAT10’s anti-anxiety and antidepressant-like effects.^369^ As one of the most abundant modifications on tRNA and rRNA, m5C is enzymatically catalyzed by members of the NOL1/NOP2/SUN domain (NSUN) family and DNA methyltransferase homolog DNMT2.^370^ By altering the translational dynamics of Glycine (Gly)-rich synaptic proteins, the impaired cortical NSUN2, which is a tRNA methyltransferase, exerts bidirectional impacts on tRNA methylation and induces depression-like behavior in the mouse brain.^371^

### Antidepressant treatments are associated with RNA modification

Altered epigenetic modification of m^6^A mRNA may contribute to therapeutic responses in psychiatric disorders. The complex protein network that maintains the m^6^A homeostasis can also be served as therapeutic target for depression potentially. Altered levels of RNA demethylase, the “erasers”, are the most frequently reported m^6^A-regulating enzymes associated with antidepressant effects. Tricyclic antidepressants increase FTO expression and activate its epigenetic function in the VTA; eliminating m^6^A modification in the VTA is postulated to cause an antidepressant effect.^372^ Moreover, activation of FTO in the hippocampus relieves depression-like behaviors and reduces the density of dendritic spines and the number of branches in mice induced by chronic restraint stress, improving the synaptic plasticity.^373^ In contrast, only a few studies reported the association between the writer and the eraser protein and effects of antidepressants. One study found that physical exercise alleviates anxiety-like behaviors and restores m^6^A levels in mouse medial prefrontal cortex; the restoration of m^6^A is related to increased expression of RNA Methylase (i.e., METTL3, METTL14, METTL16) and downregulation of RNA demethylase (i.e., ALKBH5), as well as upregulated expression of the RNA-binding factor YTHDF3.^374^ Another study showed that NAc-deep brain stimulation (DBS) could reverse the impacts of CUMS on gene expression and m^6^A-mRNA modification for certain genes, but the m^6^A-regulating enzymes were not detected.^375^

## Chromatin remodeling factor alteration is associated with MDD

Modifying chromatin structure by ATP-dependent protein complexes is also recognized as an essential epigenetic process. These protein complexes depend on ATP hydrolysis to control the histone arrangement, steering a dynamic process of DNA accessibility.^376^ ATP-dependent complexes can be classified into four groups (referring to the specificity of distinct ATPase subunits): switch/sucrose nonfermentable (SWI/SNF), imitation switch (ISWI), inositol (INO80) and chromodomain helicase DNA-binding (CHD).^376,377^ Among these, ATP-utilizing chromatin assembly and remodeling factors (ACF) are the most extensively studied protein complexes of the ISWI family. It consists of various subunits, including the bromodomain adjacent to zinc finger domain 1A (BAZ1A). Mice with ACF overexpression in NAc exhibit depression-like behaviors after social defeat stress.^378^ This study also showed that ACF overexpression regulates nucleosome architecture at transcriptional start sites, inhibiting the expression of a subset of genes, and increases susceptibility to stress-induced depression in mice and humans.^378^ The inactivation of ATPase subunit of the SWI/SNF complexes, Brahma Related Gene 1, reduces the expression of stress- and cocaine-induced immediate early genes, increases heterochromatin levels, and generally reduces chromatin accessibility in striatal medium spiny neurons.^379^ Other studies reported altered levels of selective ATP-dependent chromatin remodeling factors in mice with high anxiety-related behavior, including SNF2H in the amygdala and CHD3 and CHD5 in the ventral hippocampus.^380^ However, the impact of ATP-dependent nucleosome remodeling complexes on depression is not well-understood, and more research is needed to explore this area.

## A translational perspective on epigenetic modifications in MDD

**Fig. 7:**
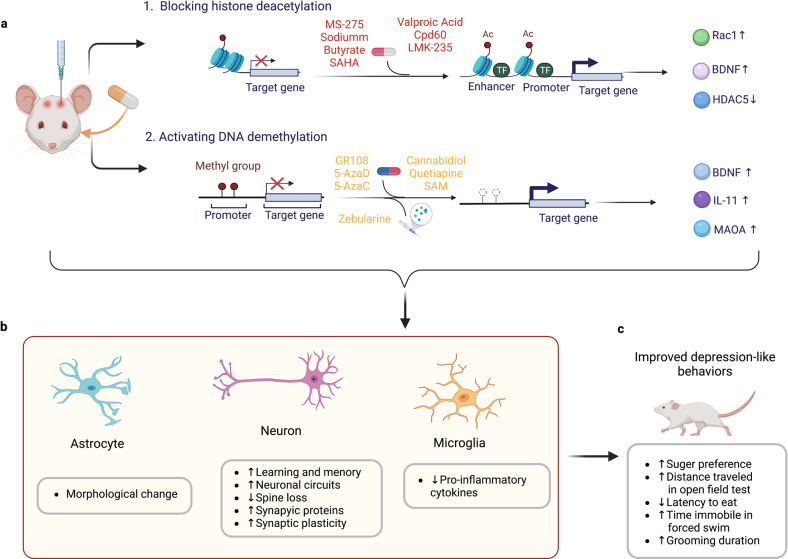
Targeting epigenetic modifications by potential antidepressants and relevant behavioral changes observed in rodents. **a** Different HDAC inhibitors, including MS-275, sodium, and butyrate can block histone deacetylation and promote the expression of target genes. DNMT inhibitors, including cannabidiol, s-adenosyl methionine and quetiapine activate DNA demethylation patterns to promote the expression of target genes. **b** These epigenetic variations lead to important recovery in the neural circuits, synaptic plasticity, astrocyte morphological changes, etc. **c** Improved depression-like behaviors in rodents after implementing drugs interfering with epigenetic mechanisms

The molecular machinery and epigenetic mechanisms related to MDD have become more understood; it is possible to test whether these epigenetic alterations have the translational potential to diagnose and aid in the treatment of MDD. Therapeutic strategies targeting writers, erasers and readers of different epigenetic components hold promise in the context of major depression and related cognitive impairments (Fig. 7).

### Biomarkers for the diagnosis of depression

Large numbers of clinical trials are currently in progress to determine whether DNA methylation, miRNAs and lncRNAs are biomarkers for different types of cancer,^381–383^ perhaps profiles of such molecules could also be used for diagnosing depression.^220,384^ It is imperative that a biomarker for the diagnosis of depression be specific for MDD, determined by whether it is a state or trait marker and whether it is affected by antidepressant treatments.^106^ Since blood samples can be collected easily, they are attractive options for diagnostic biomarkers.^385,386^ Because miRNA and lncRNA levels in blood distinguish depressed patients from healthy controls,^243,310,387^ for a diagnostic tool, it is not essential if blood and brain exhibit significant similarities.^388,389^ Results are summarized in Table 2.

**Table 2:** Summary of promising epigenetic biomarkers for diagnosis in depressed patients or depression-like animals

ncRNA	Species	Tissue	Mechanism/effects	References
miR-124↑	Human	Serum	↑miR-124 → SAT1, SMOX genes → altered synaptic plasticity	^465–469^
	Rodents	Peripheral blood mononuclear cells	↑miR-124 → BDNF-TrkB signaling pathway → inhibit neurogenesis	
		Plasma	↑miR-124 → neuronal differentiation	
		Prefrontal cortex (BA46)	↑miR-124 → synaptogenesis and neuronal proliferation;	
		Hippocampus	↑miR-124 → microglial activation	
miR-139-5p↑	Human	Prefrontal cortex (BA44)	↑miR-139-5p → inhibit neural stem cell proliferation and neuronal differentiation	^245,470^
	Rodents	Blood-derived exosome	↑miR-139-5p → target SAT1 and SMOX genes → influence neurotransmitter transmission	
		Hippocampus		
		Blood-derived exosome		
miR-221↑	Human	Prefrontal cortex (BA10)	↑miR-221 → activate the Wnt2/CREB/BDNF axis → depression symptom	^334,471–474^
	Rodents	Cerebrospinal fluid	↑ miR-221 → activate the IRF2/IFN-a pathway → depression symptom	
		Cerebrospinal fluid		
		Serum		
		Serum		
		Hippocampus		
miR-218↓	Human	Prefrontal cortex (BA44)	↓miR-218 → target Netrin-1 guidance cue receptor DCC	^266,475^
	Rodents	Plasma	↓miR-218 → regulating density of thin dendritic spines	
		Prefrontal cortex (BA44)		
		Medial prefrontal cortex		
miR-17-5p↓	Human	Locus coeruleus	↓miR-218 → target CREB1, CHRM2, NTRK3, and SLC17A7 genes	^247,476^
		Plasma		
miR-335↓	Human	Prefrontal cortex (BA9)	↓miR-335 → target GRM4, SOX4, PTPRN2, and MERTK genes	^244,477^
		Whole blood		
miR-1202↓	Human	Prefrontal cortex (BA44)	↓miR-1202 → target GRM4 gene	^340,478^
		Serum	↓miR-1202 → regulating the metabolism of glutamate	
miR-135-a↓	Human	Dorsal raphe/raphe magnus	↓miR-135-a → Serotonin transporter and serotonin receptor-1a transcripts	^479^
		Whole blood		
miR-184↓	Human	Anterior cingulate cortex	↓miR-184 → target NCOR2 and PDE4B genes	^478,480^
		Plasma		
		Serum		
miR-34c-5p↑	Humans	Peripheral blood leukocytes	↑miR-34c-5p → target SAT1, SMOX, and NOTCH1	^238,245^
		Prefrontal cortex (BA44)		
miR-24-3p↑	Human	Prefrontal cortex (BA44)	↑miR-24-3p → active MAPK/Wnt signaling pathway	^243,340^
		Whole blood		
miR-146a↓	Human	Prefrontal cortex (BA9)	↓miR-146a → TLR4 signaling pathway	^244,481^
		Peripheral blood mononuclear cells		
miR-425-3p↑	Human	Prefrontal cortex (BA44)	↑miR-425-3p → active MAPK/Wnt signaling pathway	^243,340,482^
		Whole blood		
		Peripheral blood mononuclear cells		
RP1-269M15.3↑	Human	Frontal cortex	Not reported	^483^
		Nucleus accumbens gangli		
RMRP↓	Human	Peripheral blood leukocytes	Not reported	^312^
	Mouse	Blood		
Y5↓	Human	Peripheral blood leukocytes	Not reported	^312^
MER11C↓				
PCAT1↓				
PCAT29↓				
LINC01108↑	Human	Peripheral blood cells	Not reported	^484^
LINC00998↓	Human	Peripheral blood cells	Not reported	^484^
LINC00473↓	Mouse	Prefrontal cortex (PFC) neurons	Not reported	^311^
TCONS_00019174↑	Human	PBMCs	↑ TCONS_00019174 → Regulate the phosphorylated GSK3β protein and β-catenin in the hippocampus → depression-like behaviors	^485^
ENST00000566208↑				
NONHSAG045500↑				
ENST00000517573↑				
NONHSAT034045↑				
NONHSAT142707↑				
ENST00000505825↑	Human	PBMCs	Not reported	^485^
NONHSAG017299↑				
NONHSAT078768↑				
lncRNAGm26917↑	Mouse	Hippocampus	Not reported	^345^

### Biomarkers for predicting the response to antidepressant treatment

The ideal epigenetic molecular marker for antidepressant treatment should satisfy three conditions: (1) the expression level in the blood changes significantly after taking antidepressant drugs; (2) the expression level in the brain tissue changes significantly after taking antidepressant drugs; and (3) the molecular mechanism(s) of DNAm/ncRNA-mediated antidepressant effects have been clarified. However, the key elements for a convenient blood-based biomarker, are that it shows a robust relationship to clinical response. We summarize the DNAm/ncRNAs findings in blood or brain tissue related to antidepressant response in Table 3. The findings suggest that some epigenetic marks could be helpful in predicting responses to psychotherapeutic intervention.^390–392^

**Table 3:** Promising epigenetic biomarkers for predicting antidepressant treatment

DNAm/ncRNA	Antidepressants	Species	Sample type	Antidepressant mechanism	References
BDNF Exon IV promoter	SSRI, SNRI Mirtazapine, TCA, Monoamine oxidase inhibitor	Human	Peripheral blood	DNAm at CpG-87↓ → higher nonresponse probability	^486^
BDNF Exon IV promoter	Escitalopram	Human	Peripheral blood	DNAm percentage at BDNF exon VI ↓ → higher rates of remission	^487^
SLC6A4 exon 1A	Selective serotonin reuptake inhibitor	Human	Peripheral blood	DNAm at SLC6A4 exon 1A ↓ → impaired response to antidepressant treatment	^488^
SLC6A4 promoter	Not mentioned	Human	Peripheral blood	CpG-2 methylation ↑ → higher clinical improvement with therapy	^489^
HTR1A promoter regions	Escitalopram	Human	PBMCs	DNAm at four CpG sites within the HTR1A/1B promoters ↑ → remission, DNAm at CpG-668 and CpG-1401 ↓ → poor treatment response	^490^
HTR1B promoter regions	Fluoxetine	Human	Peripheral blood	Negative association between the clinical response as assessed by GAF^3^/CGAS^4^ and the average DNAm at the HTR1B promoter	^491^
HTR1B promoter regions	Escitalopram	Human	PBMCs	DNAm at HTR1B_2 amplicon CpG-100 and HTR1B_4 amplicon CpG-1401 ↑ → remission	^490^
Interleukin-11 (IL11)	Escitalopram or nortriptyline	Human	Peripheral blood	DNAm at CpG-5 ↓ → better antidepressant response, DNAm at CpG-4 ↑ → better antidepressant response, but with worse response in those taking nortriptyline.	^492^
MAOA exon promoter	Escitalopram	Human	Peripheral blood	DNAm at CpG-1 CpG-5 ↓ → worse treatment response in females.	^493^
PPFIA4 exon I	Paroxetine	Human	Peripheral blood	DNAm within PPFIA4 ↑ → worst responders	^494^
CHN2	Escitalopram	Human	Peripheral blood	Responders showed relative decreases in both DNA methylation and mRNA expression at two CpG probes (cg23687322; cg06926818) compared to non-responders	^495^
JAK2	Escitalopram	Human	Peripheral blood	In comparison to non-responders, responders showed relative reductions in DNA methylation and mRNA expression at one CpG probe (cg08339825).	^495^
RHOJ 5ʹ-UTR and first exon	Fluoxetine	Human	Peripheral blood	RHOJ displayed four CpGs in non-responders that were noticeably hypermethylated.	^496^
OR2L13	Fluoxetine	Human	Peripheral blood	OR2L13 presented three CpGs that were significantly hypomethylated in non-responders	^496^
SORBS2	Escitalopram	Human	Peripheral blood	Seven CpG-sites in an enhancer region of SORBS2, differentially methylated between responders and non-responders, and hypermethylated in the responder group	^497^
miR-335↑	Citalopram	Human	Blood samples	miR-335↑ → inhibit GRM4 expression	^477^
miR-135↑	SSRI	Human	Blood and brain samples	miR-135↑ → regulate the serotonin transporter and serotonin receptor	^479^
miR-124↑	Citalopram	Human	Blood samples	miR-124↑ → decrease the expression of GR	^498^
	Gypenosides	Mice			
			Blood samples		
miR-155↓	Citalopram	Human	Neural progenitor cells Hippocampal neurons	miR-335↓ → increase SIRT1 expression	^499,500^
	Saikosaponin d	Rats		miR-335↓ → increase the expression of FGF2	
miR-16↑	citalopram	Rats	brain samples	miR-16↑ → decrease SERT protein levels	^330^
miR-29b-3p↑	Ketamine	Rats	Prefrontal cortex and primary neurons	miR-29b-3p↑ → decrease the expression of GRM4	^345^
miR-18↓	Kampo medicine Yokukansan	Mice	hypothalamus	miR-18↓ → regulate the expression of GR	^501^
miR-16↑	Dingzhi Xiaowan	Rats	Hippocampus	miR-16↓ → inhibit the reuptake of 5-HT	^502^
			hippocampal neurons		
miR-144-3p↓	Ketamine	Mice and human	Peripheral blood	miR-144-3p ↓ → prior to stress and following ketamine treatment in ketamine responsive mice only	^503^
hsa_circRNA_103636	Citalopram	Human	Peripheral blood	Not reported	^504^
rno_circRNA_014900	Ketamine	Rats	Hippocampus	rno_circRNA_014900 ↑, rno_circRNA_005442 ↓ →Wnt signaling, long-term depression, PI3K-Akt signaling, etc.	^505^
rno_circRNA_005442					
circDYM	Repetitive transcranial magnetic stimulation	Human	Peripheral blood	Baseline plasma circDYM levels positively correlated with the scores of depression and retardation.	^506^

### Drugs interfering with epigenetic mechanisms as potential antidepressants

Several antidepressants targeting DNA methylation are in clinical development, including quetiapine (drug repurposing), S-adenosyl methionine, and cannabidiol.

#### Quetiapine

An atypical antipsychotic, quetiapine, exerts an antidepressant effect and has the protective effect of reducing epigenetic changes induced by stress in early life. Rats with maternal deprivation showed depression-like behavior in the forced swimming test (FST), and increased activity of HDACs and DNMTs in the hippocampus and NAc. Quetiapine reversed depression-like behavior in mice and reduced DNMT activity in the hippocampal region.

#### S-adenosyl methionine

S-adenosylmethionine (SAM) serves as a methyl donor with a broad range of applicability and is occasionally administered to individuals who exhibit unresponsiveness to primary antidepressant treatments. The anti-inflammatory actions of SAM might be beneficial in treating depression. SAM controls the activation of inflammatory genes by inducing changes in DNA methylation at specific gene promoters. SAM reduces the expression of the proinflammatory cytokine TNFa and the chemoattractant CCL2 and its receptor CCR2, in association with DNA methylation changes in specific gene promotor.^393^ In contrast, DNA methylation by SAM increases IL-10 expression, an anti-inflammatory cytokine.^393^

#### Cannabidiol

Cannabidiol (CBD) is cannabis’ most abundant non-psychoactive component.^394^ CBD’s therapeutic potential for reducing depression-like behavior has been shown, since it interacts with a wide range of brain molecules^395,396^. In one study,^397^ stress induced by FST increased levels of global DNA methylation levels and DNMT activity in the hippocampus while decreasing DNA methylation and DNMT activity in the prefrontal cortex. Interestingly, CBD administration prevented stress-induced epigenetic changes in both the hippocampus and PFC.^397^ A preliminary study reported that several histone modifications were changed in some brain regions of rats after CBD administration.^398^ But the molecular mechanism of CBD action remains to be investigated.

#### DNMT inhibitor

Different DNMT inhibitors (DNMTi) inhibit DNMTs through different mechanisms, including nucleosides and nonnucleosides. Nucleosidic DNMTi, including 5-AzaC, 5-AzaD, and zebularine, are chemical analogs of cytidine, which are integrated into DNA molecules during replication (S-phase). Their covalent binding to the DNMTs prevents DNA methylation and irreversibly blocks these enzymes.^399^ It has been demonstrated that 5-AzaD and 5-AzaC lead to a decrease in DNA methylation and increased BDNF expression in the hippocampus, displaying effects similar to those of antidepressants in several preclinical settings.^400^ In addition, injecting zebularine into the cerebral ventricles of adult rats for seven days reduced DNA methylation of the BDNF promoter.^401^ The reduced methylation of the promoter enabled this drug regimen to restore BDNF expression, suggesting the potential of zebularine as a therapeutic agent in clinical use. Some nonnucleoside inhibitors, such as RG108 and procaine, have different mechanisms of action, including noncovalent inhibition within the DNA catalytic sites, to impede enzymatic activity. RG108 alters stress-induced DNA methylation bidirectionally in the hippocampus and PFC and triggers antidepressant-like effects as demonstrated in the forced swimming test.^402^

The therapeutic potential of HDAC inhibitors (HDACis) is suggested because HDACis may augment the efficacy of antipsychotic medications on coadministration.^403,404^ Animal models and postmortem brain results show some HDACis exert antidepressant-like effects (Table 4, Fig. 7). HDACis, such as sodium butyrate and SAHA, promote cognitive function, and may provide therapeutic options for depressed patients with cognitive impairment.^405–407^ Although medications targeting these epigenetic molecules are proving effective in preclinical animal studies, there is a lack of successful clinical trials of HDACi in neuropsychiatric disorders, perhaps because of the complexity of different HDAC isoforms (i.e., HDAC2 and HDAC5) that are associated with pro-depressant and antidepressant functions.^408^ The complexity is further heightened because HDAC and DNMT are widely distributed in the CNS and peripheral tissues. Interference in nonneuronal or inappropriate neuronal locations may induce side effects and off-target effects.^409^ Drugs that target brain tissue-specific epigenetic modulators may ultimately prove to be helpful new therapies for depression, but the studies that show this remain to be done.

**Table 4:** Drugs targeting epigenetic processes as a therapeutic strategy for depression

Target	Drug	Species	Molecular Mechanisms of Action	Reference
Class I HDAC inhibitor	MS-275	Mice	↑H3 acetylation in the mPFC, exerting antidepressant-like effects	^507^
			↑Rac1 in the NAc, synapse structural plasticity normalization	^158^
			↑H3 acetylation in the hippocampus and the NAc, exerting antidepressant-like effects	^127,130^
Class I and II HDAC inhibitor	Sodium butyrate	Mice	HDAC5 downregulation, ↑H3 acetylation in BDNF gene promoter, ↓depression-like behavior	^148^
			↓Depression-like behavior, ↑HDAC2, ↑pCREB, ↑H3 acetylation, ↑BDNF in the hippocampus	^508^
		Rats	↓Depression-like behavior, ↑transthyretin (Ttr) expression, ↓serotonin 2A receptor, ↑H4 acetylation at Ttr gene promoter	^129^
	SAHA	Mice	↑H3 acetylation in the hippocampus and the NAc, exerting antidepressant-like effects,	^130^
			↑Gdnf in the NAc, HDAC2 inhibition	^104^
			↓Depression-like behavior, ↑BDNF in the PFC	^509^
	Valproic Acid	Rats	↓Depression-like behavior, ↓corticosterone plasma level	^510^
HDAC1/2 inhibitor	Cpd60	Mice	↑Histone acetylation at the promoter regions of upregulated transcripts	^511^
HDAC4/5 inhibitor	LMK-235	Mice	↓Depression-like behavior	^469^
SIRT2 inhibitor	33i	Mice	↑Serotonin levels, ↑glutamate receptor subunit expression, ↓depression-like behavior	^512^
SIRT1 activator	SRT2104	Mice	Exerting antidepressant-like effects, ↓spine loss, ↑phosphorylation level of protein kinases 1 and 2 in the stressed condition	^151^
DNMT inhibitor	Zebularine, RG108	Mice	Exerting antidepressant-like effects, ↓DMNT at Gdnf promoter	^104^
	RG108		Exerting antidepressant-like effects	^99^

## Concluding remarks and future directions

Environmental stressors and genetic factors shape the complex phenotype of MDD. Specifically, epigenetic processes shape and store a cell’s molecular response to its environment. The prevailing hypothesis suggests that epigenetic processes leave imprints on the genome, which then interact with an individual’s genetic predisposition to determine their susceptibility to depression over the course of their lifetime. Therefore, exploring epigenetic mechanisms provides fresh perspectives on the pathophysiology of MDD and has the potential to generate innovative biomarkers for diagnosis and treatment purposes. Established animal models for depression and video-recorded behavior analyses have greatly advanced the understanding of various epigenetic mechanisms contributing to stress responses, neuroplasticity, neurotransmission, and neuroglial function. Clinical studies have focused on DNA methylation and ncRNA using human peripheral blood samples, with few focusing on histone modifications.^410^ As MDD is a stress-related neuropsychiatric disorder, validating the employment of peripheral epigenetic molecular alterations as surrogates for what occurs in the brain does not advance our knowledge of pathogenesis. Still, it may be useful in developing biomarkers of disease and treatment response.^98^

Innovative approaches are necessary to enhance our understanding of the pathogenesis in the brain. Fluorescence-activated cell sorting techniques and single-cell epigenomics can facilitate the examination of DNA methylation various cell types.^106,411^ Likewise, although the patterns of histone modification usually differ between cell types, most studies conducted have examined these modifications in cell populations that are heterogeneous. A high priority for future studies is to probe the epigenetic landscape by integrating modern low and single-cell technologies in combination with next-generation sequencing, to simultaneously measure multiple molecules such as proteins and RNAs, DNA methylation, or chromatin accessibility.^412,413^

Some types of epigenetic modifications, such as changes in chromosome conformation are still largely unexplored in MDD.^414,415^ The chromosome conformation capture (Hi-C) technique can provide information on the 3D chromosome conformation and uncover the interactions between distant genomic regions that affect gene expression. The transposase-accessible chromatin sequencing (ATAC-Seq) assay is also a powerful tool for investigating genome-wide chromatin accessibility.^416,417^ Recent MeRIP-Seq methods map the m^6^A-methylated RNA^418^, offering a new way to explore the transcriptome-wide location of m^6^A in MDD. Combined with the widely used next-generation RNA sequencing techniques, these techniques for exploring the epigenome may facilitate a more comprehensive understanding of the possible mechanisms that contribute to MDD.

Epigenetic processes require a pool of metabolites, such as acetyl-CoA for histone acetylation and S-adenosyl-methionine for methylation. Recent studies provide evidence for metabolic signaling to chromatin in the brain.^419^ Lactate-dependent histone lactylation (H4K12a) in microglia promotes the expression of glycolytic genes, leading to microglial dysfunction and cognitive decline.^420^ Additional research is required to investigate epigenetic pathways that are dependent on metabolism, in the pathogenesis of depression and its potential to mimic the effects of antidepressants. In addition, several studies have established an association between the gut microbiome, the metabolites of the gut flora and epigenetics, revealing the impact of the gut microbiota on ncRNA production.^421,422^ Extensive evidence demonstrates that the brain-gut-microbiota axis plays a crucial role in the pathophysiology of MDD and other psychiatric disorders.^423,424^ Hence, investigating the effects of the combination of the gut microbiome and epigenetic modification could potentially shed light on the underlying mechanisms of depression.

Finally, manipulating targeted epigenetic states is crucial to gain a comprehensive understanding of the effects of stress-induced histone mark alterations. As mentioned above, the clinical adaptation of HDAC/DNMT inhibitors is severely constrained by their off-target effects. For example, the true causality of epigenetic signatures at DNA sites that are known to be involved in stress-related disorders can only be examined with the advent of novel mutagenesis tools such as Clustered Regularly Interspaced Short Palindromic Repeats (CRISPR-Cas9) and Transcription Activator-Like Effector Nucleases (TALENs).^425,426^ These technologies facilitate the targeting of epigenetic modifiers to specific genes, brain regions, and time frames to ascertain whether the regulation of epigenetic modifications at a particular locus is accountable for a certain psychiatric disorders.^427^ The use of CRISPR/Cas9 system enables regulation of gene expression and make epigenetic alterations without introducing DNA double-strand breaks.^428^ Furthermore, CRISPR-cas9 system can trans-epigenetic remodel the endogenous gene.^429^ Recently, a new set of epigenetic editor CRISPRoff has been developed, enabling persistent, heritable, and reversible DNA methylation modification and regulation of gene transcription.^430^ These tools specifically allow the targeting, cutting, and replacing a specific epigenetic-related gene to explore the gene functions in psychiatric disorders. It is believed leveraging these tools can help elucidate more potential epigenetic mechanisms in MDD.
